# Metagenomic insights into oral microbiota dynamics in diabetic and non-diabetic periodontal disease: a pilot study

**DOI:** 10.3389/fmicb.2026.1799124

**Published:** 2026-04-14

**Authors:** Harshvardhan, Mahaldeep Kaur, Vishakha Grover, Anil Kumar Pinnaka, Suresh Korpole

**Affiliations:** 1 Microbial Type Culture Collection and Gene bank, CSIR-Institute of Microbial Technology, Chandigarh, India; 2 Academy of Scientific and Innovative Research (AcSIR), Ghaziabad, India; 3 Dr. Harvansh Singh Judge Institute of Dental Sciences and Hospital, Panjab University, Chandigarh, India

**Keywords:** dysbiosis, metagenomics, oral microbiome, pathogen, periodontitis, probiotic, subgingival plaque

## Abstract

**Introduction:**

Subgingival microbial dysbiosis is one of the key reasons behind periodontitis, a chronic inflammatory disease, which is further get severe in the presence of type 2 diabetes mellitus (T2D). Although changes in taxonomic composition have been well established, the functional interactions and metagenomic profiles across different stages of the disease remain unclear.

**Methods:**

A shotgun metagenomic analysis was performed on subgingival dental plaque samples from 16 individuals, divided into healthy, staged periodontitis, and diabetic periodontitis groups. Group-wise DNA pooling was done for maximum DNA yield. Further, Alpha/beta diversity, taxonomic profiling, pathogen-probiotic ratios, and metabolic pathway abundance were analyzed and studied.

**Results:**

The healthy group showed the highest alpha diversity, especially in the core biosynthetic pathways. On the other hand, the earlier stages of periodontitis showed a unique community structure and the lowest alpha diversity. Early periodontitis also showed the highest abundance of commensals like *Actinomyces* and *Bifidobacterium*, along with increased UMP/guanosine and L-arginine biosynthesis pathways. The advanced periodontitis group had an increase of red complex bacteria and loss of probiotics. An increase of the degradative pathways, such as L-histidine degradation, had also been observed in this stage. The diabetic periodontitis group had a distinct microbial profile that included *Capnocytophaga* and a considerable metabolic shift toward lipid metabolism and glycolysis, with higher overall microbial diversity than the other periodontitis groups.

**Conclusion:**

The results clearly show that the subgingival microbial and functional patterns are different across the stages of the disease and metabolic status, which can be developed for underscoring the importance of targeting early metabolic shifts to prevent dysbiosis.

## 1 Introduction

The oral cavity harbors one of the most diverse microbial ecosystems in the human body, which includes the tongue dorsum, buccal mucosa and the hard structure of teeth, comprising both subgingival (below) and supragingival (above) niches (The Human Microbiome Project Consortium, 2012; Xu et al., 2015). The oral cavity comprises over 700 bacterial species contributing to a complex and dynamic oral microbiome (Deo and Deshmukh, 2019). This microbiome plays a crucial role in maintaining oral health by competitively excluding pathogens, modulating the immune system, and regulating metabolism (Lamont et al., 2018). In a balanced state (eubiosis), commensal bacteria such as *Streptococcus, Actinomyces*, and *Veillonella* dominate, forming a protective biofilm that promotes the ecological stability (Radaic and Kapila, 2021). Disruption of this balance or dysbiosis causes excessive host immune responses leading to periodontal tissue degradation, alveolar bone loss and ultimately periodontitis (Lamont et al., 2018). It is the most common chronic infectious disease of the periodontal tissues and a leading cause of tooth loss in adults. Globally, severe periodontitis affects nearly 10% of adults highlighting the widespread impact of this disease (Frencken et al., 2017). Periodontitis progresses through stages, each with distinct microbial and clinical characteristics. Early stages may elicit compensatory increases in beneficial microbes and heightened metabolic activity, whereas advanced diseases are associated with functional decline and the expansion of classical pathogen (Tonetti et al., 2018). Further, systemic ailments like type 2 diabetes (T2D) are closely linked to periodontitis in which the relationship goes both ways, making each condition worse (Sanz et al., 2018; Shi et al., 2023). 16S rRNA gene-sequence-based studies were reported on taxonomic changes in the subgingival microbiome during health, periodontitis, and diabetes with focus on genera-level resolution and do not directly address functional metabolic phenotypes (Huang et al., 2021; Wang et al., 2013). Further, shotgun metagenomic analyses have started to identify functional similarities in periodontitis and peri-implantitis (Wang et al., 2013; Huang et al., 2021), but they were largely focused on comparison between health and a single chronic disease condition without distinguishing between early and advanced periodontitis or diabetic and non-diabetic disease conditions. Similarly, metagenomic studies demonstrated that T2D periodontitis has a unique community and pathway profile, but again focused on comparison between diabetic and non-diabetic conditions without distinguishing between the various clinical stages (Shi et al., 2020; Lu et al., 2022). Recent reviews underscore lack in stage-resolved understanding of the pathogen/symbiont ratio, and metabolic function changes in different stages of periodontitis including diabetic condition (Huang et al., 2021; Siddiqui et al., 2023).

Shotgun metagenomic sequencing not only provides species or strain resolution but also functional gene and pathway annotation, and thereby allowing to study community structure and function in a single analytical framework (Quince et al., 2017; Beghini et al., 2021). Thus, it overcomes the limitation of 16S rRNA gene-based profiling. However, there is a lack of shotgun studies on clinical staging and diabetic stratification in subgingival plaque samples, especially, in resource-poor settings where the cost of sequencing is a barrier to entry (Lu et al., 2022). Thus, in this pilot study, shotgun metagenomics of pooled subgingival plaques from four well-stratified clinical stages including healthy periodontium, early periodontitis (Stage I/II), advanced periodontitis (Stage III/IV), and diabetic periodontitis (Stage III/IV with T2D), was used to understand alpha and beta diversity, taxonomic composition, including abundant and rare species to constitute a core versus stage-restricted subgingival microbiome. Also, we made an attempt to throw light upon major metabolic functions, especially, metabolic change with advancing periodontal disease and in diabetes. By exploiting a cost-efficient pooled library design, (Teufel and Sobetzko, 2022) this study is a pilot-scale, pooled-design exploratory study, which offers exploratory, quantitative hypotheses that to be validated in larger individual-level datasets.

## 2 Material and methods

### 2.1 Study design

Dental subgingival plaque samples were collected as a part of hospital based observational study involving participants diagnosed with periodontitis, diabetic periodontitis and healthy controls. The Institutional Ethics Committee of Panjab University, Chandigarh (PUIEC) approved the study protocol and all procedures adhered to the Declaration of Helsinki. Each adult participant who was included in the study at the Department of Periodontology, Dr. H.S. Judge Institute of Dental Sciences & Hospital, Panjab University, Chandigarh, signed an informed consent form. A total of 16 participants were enrolled with early-stage periodontitis (*n*=4; Stage I–II), advanced-periodontitis (*n*=4; Stage III–IV), diabetic periodontitis (n=4 periodontitis patients with type II diabetes mellitus) and healthy individuals (n=4 absence of periodontal disease and diabetes). The inclusion criteria included age (26-52 years), presence of at least 20 natural teeth, completion of a comprehensive medical and oral examination, and willingness to participate. The overall age range of 26-52 years was intentionally selected for typical adult-onset periodontitis. It is pertinent to mention that under 25 years of age minimizes any confounding effects of rapid-grade, early-onset periodontitis, which is characterized by a distinct and highly virulent microbial profile (Thomas et al., 2025). Similarly, restricting the maximum age to 52 years avoids any confounding effects of age-related immunosenescence, polypharmacy, and edentulism, all of which have profound and independent effects on the oral microbiome (Feres et al., 2016). The exclusion criteria included pregnancy or lactation, current smoking or tobacco use, systemic antibiotic or anti-inflammatory therapy within 1 month prior to sampling. Further, periodontal therapy within the preceding six months, other systemic conditions known to affect periodontal status (e.g., immunodeficiency, malignancy), and inability to provide informed consent (Sanz et al., 2018) were also considered in exclusion criteria. Additional parameters such as sex, body mass index (BMI), and medical history (hypertension, diabetes status, alcohol consumption) were also recorded (Supplementary Table 1).

### 2.2 Clinical oral examination

Oral examinations were performed between August 2021 and January 2022 at Dr. HSJ Institute of Dental Sciences and Hospital. Before the study, intra-examiner calibration was conducted to evaluate the reproducibility of periodontal parameters. All periodontal clinical recordings were done by a single, trained examiner. Fifteen percent of the periodontal sites (50 sites), randomly selected from individuals (not included in the study), were re-assessed after 1 week, under the same clinical conditions, to evaluate intra-examiner reproducibility. The examiner was unaware of the initial recordings during the re-assessment. Bleeding on probing (BOP) was scored as a binary variable, i.e., the absence or presence of bleeding on probing. The probing pocket depth (PPD) and clinical attachment level (CAL) were evaluated using a periodontal probe and scored using ordinal categories (PPD: ≤ 3 mm, 4-5 mm, ≥6 mm; CAL: 0-2 mm, 3-4 mm, ≥5 mm). The intra-examiner reproducibility of BOP was evaluated using Cohen's kappa statistic, and weighted kappa with linear weighting was used to evaluate the intra-examiner reproducibility of PPD and CAL to adjust for ordinal measurement differences (Cohen, 1960). The examiner showed high reproducibility, with almost perfect agreement for all parameters (BOP κ = 0.88; PPD weighted κ = 0.88; CAL weighted κ = 0.85), thus ensuring proper calibration before starting the study. Kappa values >0.80 indicate excellent intra-examiner agreement.

### 2.3 Samples collection

Diseased sites were selected as per standard criteria (Tonetti et al., 2018). Clinical parameters including gingival condition, PPD, BOP, suppuration, presence of plaque and CAL values and radiographic bone loss and tooth loss were assessed. Healthy subjects had PPD ≤ 3 mm, no CAL, no radiographic bone loss, and no history of diabetes. Early and advanced periodontitis groups had CAL and radiographic bone loss within stage criteria, while the diabetic periodontitis group had physician-diagnosed T2D with HbA1c ≥6.5% (Sanz et al., 2018; Lu et al., 2022). Subgingival plaque samples were collected from multiple sites (using 3-4 sterile paper points per site) within each patient's mouth, with the paper points left undisturbed in the gingival sulcus for approximately 20 s. The subgingival plaque samples from multiple sites within each patient were then pooled together, and immediately stored in phosphate-buffered saline (PBS) containing 50 mM dithiothreitol (DTT) to dislodge biofilm material for DNA extraction. Demographic and clinical variables recorded for each participant included age, sex, body mass index (BMI), smoking status, alcohol consumption, diabetes and hypertension status, recent antibiotic use, BOP, PPD, CAL, caries, and number of missing teeth (Table 1; Supplementary Table 1).

**Table 1:** Baseline demographic and clinical characteristics of study groups.

Clinical Group	*n*	Age (Mean ±SD)	Sex (M/F)	BMI (Mean ±SD)	Mean BOP (%)	Mean PPD (mm)	Mean CAL (mm)	HbA1c (%)
**Healthy**	4	26.8 ± 0.96	0/4	21.0 ± 1.63	11.75	1.75	0.00	-
**Early Periodontitis (Stage I/II)**	4	35.0 ± 4.69	2/2	25.75 ± 3.30	30.00	3.25	3.50	-
**Advanced Periodontitis (Stage III/IV)**	4	46.0 ± 4.97	3/1	31.0 ± 2.58	60.50	8.25	7.00	-
**Diabetic Periodontitis**	4	46.0 ± 5.23	2/2	27.5 ± 2.52	57.75	8.25	8.50	6.5–11.0

### 2.4 DNA extraction and metagenomic sequencing

Metagenomic DNA was isolated from human dental subgingival plaque samples using the Zymo Quick-DNA Fungal/Bacterial Miniprep Kit (Zymo Research). Due to scanty biomass in each subgingival sample, DNA isolated from the four subjects in each clinical group was pooled after extraction to create one pooled library per group. An equal amount (~1μg) of high-quality DNA from each subject was mixed to contribute equally to the pool. The method of creating a pooled library has been demonstrated to improve DNA concentration and lower library preparation and sequencing expenses in exploratory metagenomic analyses without diminishing key taxonomic and functional information (Teufel and Sobetzko, 2022). Following extraction, quantification of DNA was performed and assessed for quality to ensure suitability for next-generation sequencing. Libraries were prepared using KAPA HyperPlus Kit (Roche, catalog no. KK8512). This method is optimized for Illumina sequencing platforms and is compatible with DNA inputs ranging from 1 ng to 1 μg, providing flexibility for various subgingival sample types. Paired end (2 × 150 bp) sequencing was performed on an Illumina NovaSeq 6,000 platform (Nucleome Informatics Pvt. Ltd. Hyderabad, India).

### 2.5 Sequence pre-processing and quality control

Raw sequencing reads were quality assessed using FastQC (v0.12.0) (Andrews, 2010). Adapters and low-quality bases were trimmed using FastP (v0.23.2) (Chen et al., 2018). Human-derived sequences were removed by aligning against the human genome (GRCh38) using bowtie2 (Langmead and Salzberg, 2012). All raw reads were submitted under NCBI SRA bioproject accession PRJNA1333333.

### 2.6 Bioinformatic analysis

In this pilot study, combined pooled metagenomic libraries from four clinical categories-healthy, early periodontitis (Stage I/II), advanced periodontitis (Stage III/IV), and diabetic (T2D) periodontitis were analyzed using a pooled-design exploratory metagenomics technique (Teufel and Sobetzko, 2022). Because of this pooling technique, variation among individuals was not evaluated, and the findings should be considered as descriptive group-level variations (Teufel and Sobetzko, 2022).

### 2.7 Taxonomic and diversity quantification

Filtered sequencing reads were processed for taxonomic profiling using Kraken2 (v2.0.8-beta) against the MiniKraken2 database, followed by Bracken (v3.0) for accurate species-level abundance estimation using default parameters (Wood et al., 2019; Lu et al., 2017). Alpha diversity indices were computed directly from species-level Bracken abundance tables per pooled subgingival sample and tabulated using standard metrics: Shannon index (H'), measuring species richness and evenness, and Simpson index (D), measuring dominance (Shannon, 1948; Simpson, 1949). Both indices were calculated as dimensionless measures without statistical hypothesis testing due to the pooled-design limitation of n=1 library per group. Community dissimilarities among categories were quantified using two complementary distance metrics: (1) Bray–Curtis's dissimilarity, accounting for both presence/absence and abundance, visualized via NMDS (Non-metric Multidimensional Scaling) and PCoA (Principal Coordinates Analysis), and (2) Jaccard distance, considering only species presence/absence. Distances were computed using SciPy (Python), saved as distance matrices, and visualized as hierarchical clustering heatmaps. These analyses were performed to illustrate ecological community structure and separation among clinical groups.

### 2.8 Visualization and relative abundance analysis

Alpha and beta diversity plots were generated using Python (matplotlib/seaborn with non-GUI backend). For genera and species-level relative abundance profiling, stacked bar plots and genera/species-level heatmaps were generated using R (v4.5.0) with ggplot2 and viridis packages for consistent visualization across clinical groups. CZID pathogen analysis was used to locate the shifts in pathogen abundance using bracken outputs (Kalantar et al., 2020). Probiotic taxa were identified by cross-referencing Bracken species-level results with the curated MetaProbiotics Global Probiotics Set (Wu et al., 2024).

### 2.9 Rare biosphere and core microbiome analysis

Gamma diversity (γ) was quantified as species richness, defined as the total number of unique species observed across all four clinical groups combined. Using this process followed by Whittaker's multiplicative α/β/γ partitioning framework using species presence/absence data extracted from Bracken tables. UpSet plots were generated using the UpSetPlot Python library (v0.9.0) to visualize shared and unique species across clinical groups (Lex et al., 2014). Species were classified into two abundance categories: abundant species (≥0.1% relative abundance in at least one group) and rare species (< 0.1% relative abundance) (Pascoal et al., 2025). Within the gamma diversity set, overlapping species patterns were analyzed to distinguish the ‘rare biosphere' (low-abundance, high-diversity organisms) from dominant taxa based on higher numbers, providing ecological context for understanding community assembly across disease stages.

### 2.10 Functional metabolic profiling

Taxonomic and functional profiling was performed with the HUMAnN v3.0.1 pipeline, and sequence alignment was performed with MetaPhlAn v4.1.1, DIAMOND v2.0.15, and Bowtie2 v2.5.1 (Beghini et al., 2021; Blanco-Míguez et al., 2023). Here, the study focused on the main pathways that were contributing to >90% of overall pathway abundance per group while reducing duplication. Functional gene abundance was assessed through pathway-level analysis rather than individual gene statistics. Metabolic pathway abundances were normalized as Reads Per Kilobase (RPK): calculated as (number of reads mapping to a pathway) / (pathway length in kilobases) (Wang et al., 2013). RPK values are presented as descriptive abundance metrics for comparative visualization across clinical groups, not as statistically tested differences. In R (v4.5.0), data were visualized as a Sankey diagram using networkD3 v0.4 (Platzer et al., 2018).

### 2.11 Data interpretation framework

Given the pooled design, which yielded *n* = 1 mixed sample per clinical category, all presented data are aggregate group-level patterns indicating the combined microbial profiles of people in each category. Inter-individual variability within each group cannot be measured. The findings should be seen as an exploratory characterization of each clinical category's microbiota and metabolic capacity, producing hypotheses and quantitative indicators for validation in future bigger, individual-level research.

## 3 Results

### 3.1 Participant demographics and clinical characteristics

Baseline demographic and clinical characteristics of the study participants are summarized in Table 1. The mean age increased progressively from healthy controls (26.8 ±0.96 years) to advanced (Stage III/IV; 46.0 ± 4.97 years) and diabetic periodontitis (46.0 ± 5.23 years) groups. Clinical parameters such as PPD, BOP and CAL values showed a stage wise increase (Table 1). The diabetic periodontitis group was confirmed to have poor glycemic control, with HbA1c levels ranging from 6.5% to 11.0%. No participants reported recent antibiotic use.

### 3.2 Diversity analyses of microbial communities across clinical groups

**Figure 1:**
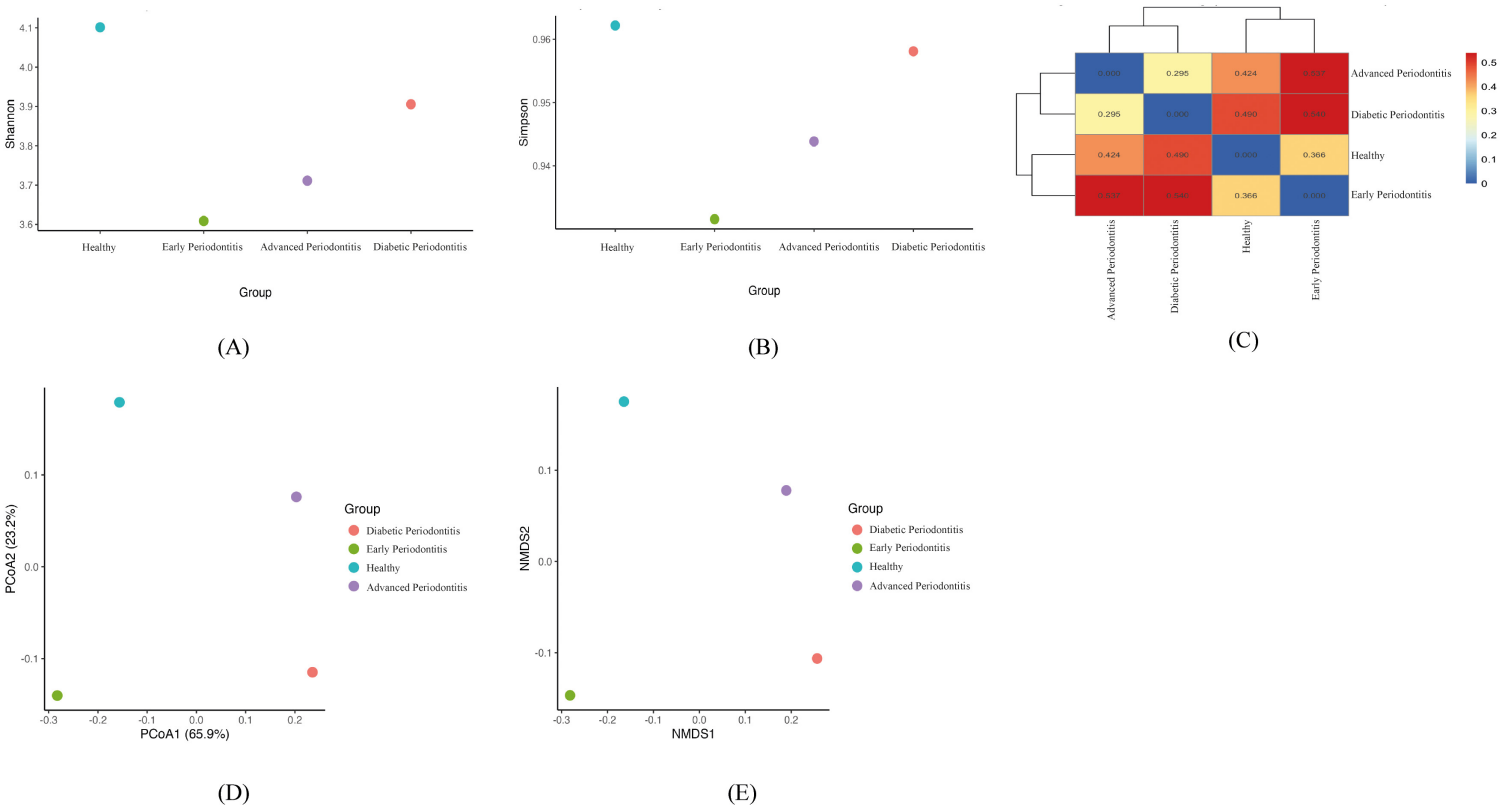
Alpha and Beta diversity analyses of microbial communities across clinical groups: Plots comparing **(A)** Shannon diversity (richness and evenness of species) and **(B)** Simpson index across the four groups: Healthy, early periodontitis, advanced periodontitis, and diabetic periodontitis. **(C)** Hierarchical Clustering Heatmap (Bray-Curtis Dissimilarity) between subgingival plaque sample groups. Colors indicate pairwise dissimilarity values; lower values (blue) indicate similar community composition. **(D)** Non-metric multidimensional scaling (NMDS) plot of oral microbiome profiles using Bray–Curtis distances. Each point represents a pooled group; clustering indicates community divergence, and **(E)** Principal Coordinates Analysis (PCoA) based on Bray-Curtis's dissimilarity further demonstrates group-wise dissimilarity in microbial composition.

Alpha diversity metrics like Shannon index and Simpson index revealed significant differences in microbial richness and diversity across the clinical groups. The healthy group showed the highest richness of species in Shannon index (Figure 1A) and also by Simpson index (Figure 1B), followed by diabetic periodontitis and advanced periodontitis, whereas early periodontitis subgingival sample displayed the lowest diversity. Beta diversity analyses performed using dissimilarity metrics showed distinct microbial community shifts. The heatmap (Figure 1C) showed the least dissimilarity between healthy and early periodontitis and the highest between diabetic periodontitis and advanced periodontitis. NMDS and PCoA plots (Figures 1D, E) showed clear, non-overlapping clusters for each clinical group, indicating that there are distinct microbial communities established by disease stage and diabetic status.

### 3.3 Metagenomic based taxonomic insights

**Figure 2:**
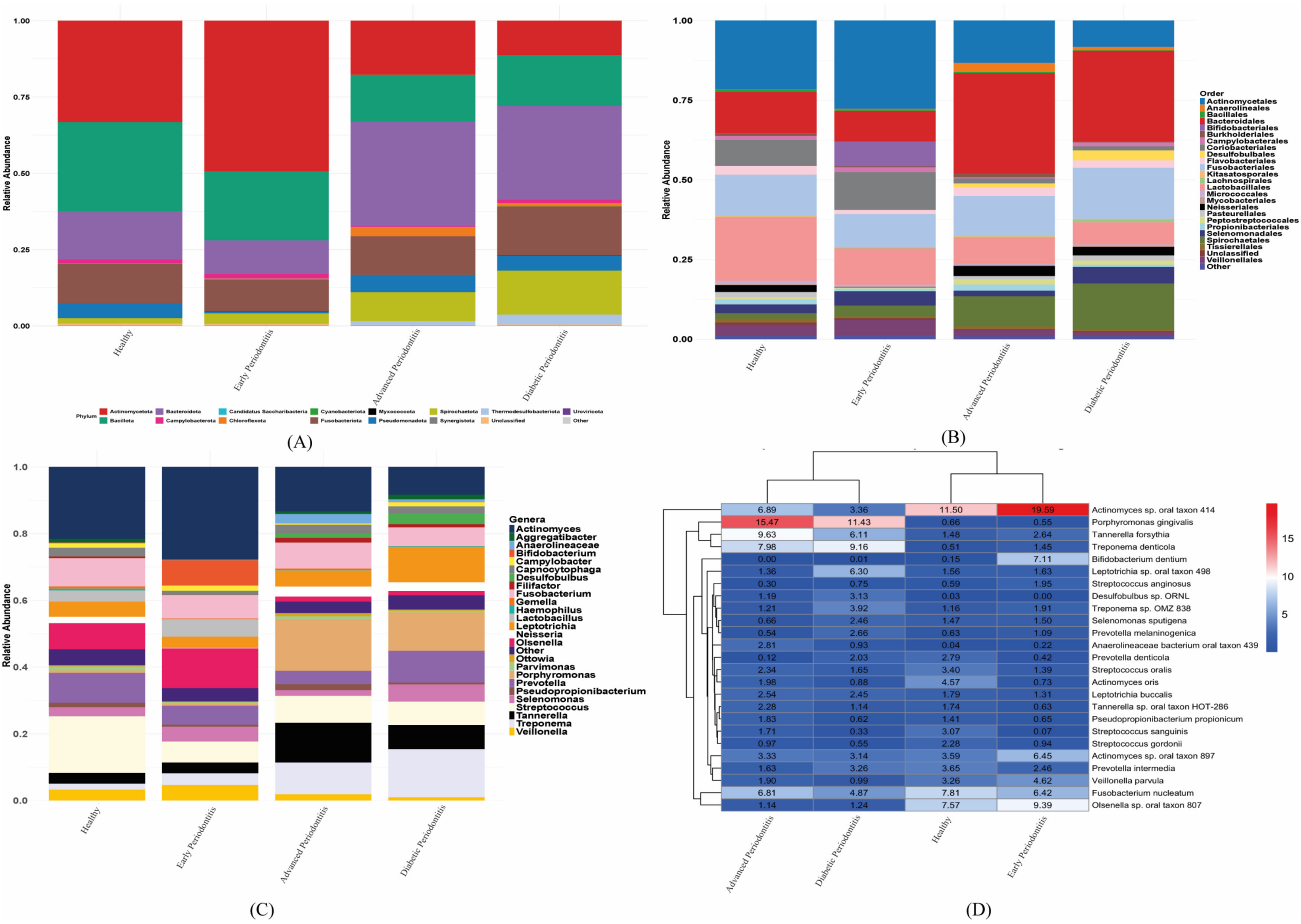
Taxonomic profiling of the oral microbiome across clinical groups. **(A–C)** Stacked bar plots showing relative abundances at the **(A)** phylum, **(B)** order, and **(C)** genera levels across the healthy, early periodontitis, advanced periodontitis, and diabetic periodontitis groups. **(D)** Heatmap of the top 25 most abundant species with hierarchical clustering reveals distinct microbial signatures, with red complex pathogens enriched in advanced disease and commensals dominant in health-associated subgingival plaque samples.

Taxonomic analysis showed increasing dysbiosis across disease stages from healthy to advanced periodontal disease (Figure 2). At the phylum level, healthy and early periodontitis subgingival samples displayed higher proportions of *Actinomycetota* and *Bacillota* (>60% combined abundance). The samples obtained from advanced periodontitis and diabetic periodontitis showed an abundance of strains from bacterial phyla such as *Bacteroidota, Chloroflexota, Spirochaetota*, and *Fusobacteriota* (Figure 2A). At the order-level, healthy samples were enriched in *Actinomycetales, Bifidobacteriales*, and *Lactobacillales* (Figure 2B), early periodontitis samples displayed a minor increase in *Bifidobacteriales, Coriobacteriales, Lactobacillales*, and *Veillonellales* (Figure 2B), while advanced periodontitis showed a higher proportion of *Bacteroidales* and *Spirochaetales*. In contrast, a reduction in health-associated orders like *Actinomycetales* and *Lactobacillales* was observed in advanced periodontitis (Figure 2B). Additionally, the progressive dysbiosis was also observed at the genera level across the clinical groups. It showed a similar shift from healthy to disease-associated bacterial profiles as the genera *Streptococcus, Bifidobacterium, Lactobacillus, Veillonella, Actinomyces*, and *Gemella* were predominated (Figure 2C). Similarly, early periodontitis showed an increased level of *Actinomyces, Bifidobacterium, Lactobacillus, Olsenella and Veillonella*. However, advanced and diabetic periodontitis showed considerable increase in abundance of pathogens belonging to genera *Treponema, Tannerella, Porphyromonas* and *Prevotella*. Furthermore, taxonomic profiling at species level showed some substantial changes in microbial community across different clinical groups. Healthy samples showed enrichment in commensal strains like *Actinomyces* spp., *Olsenella* spp., *Fusobacterium nucleatum, Prevotella intermedia* and *Streptococcus oralis* (Figure 2D). Early periodontitis also showed increase in *Actinomyces* spp., *Olsenella* spp., *Bifidobacterium dentium, Tannerella forsythia, Treponema denticola*, and *Streptococcus anginosus*. Whereas, advanced and diabetic periodontitis were predominated with *P. gingivalis, T. forsythia*, and *T. denticola*.

### 3.4 Abundant and rare species analyses

**Figure 3:**
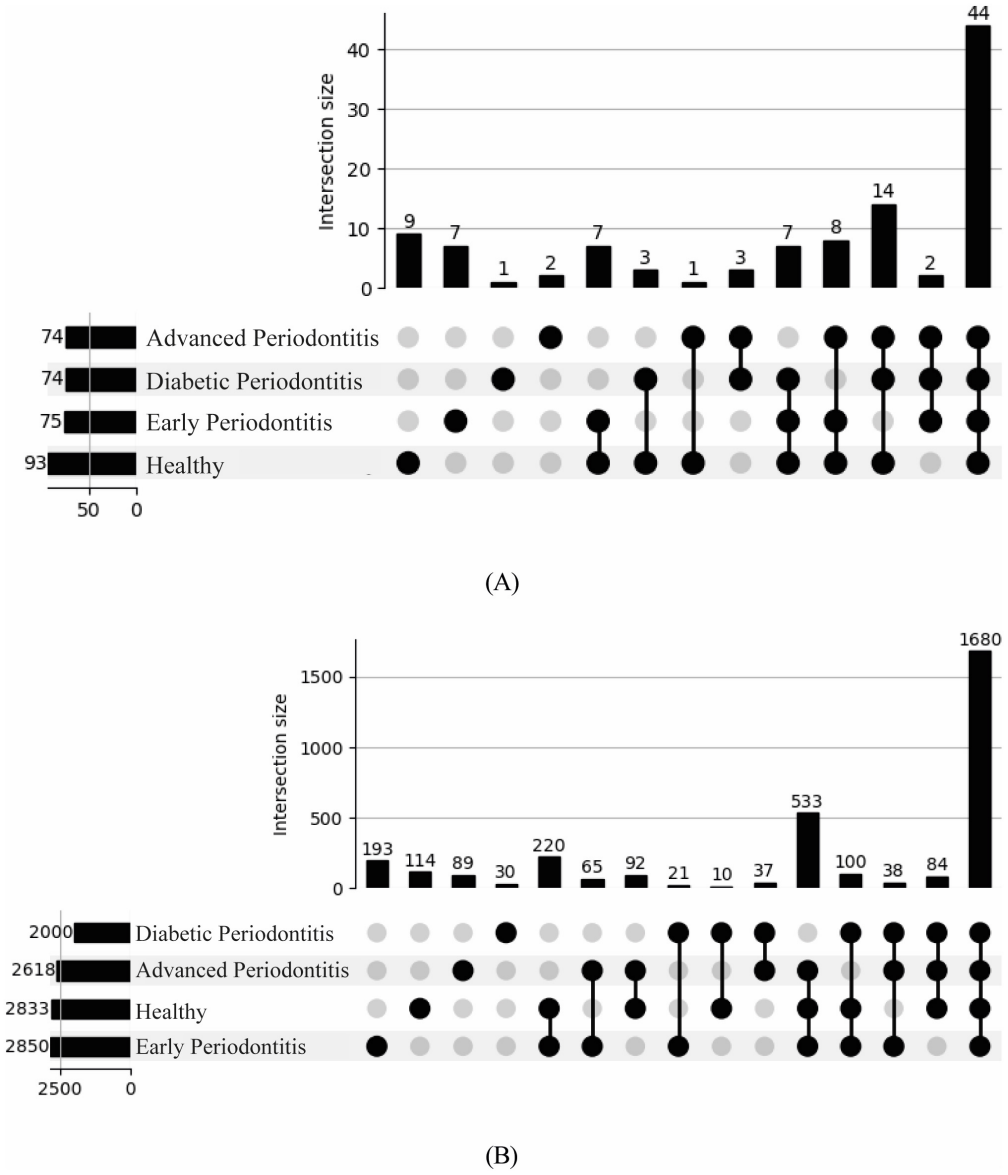
Upset plots depicting shared and unique species across clinical groups: **(A)** Abundant species and **(B)** Rare species.

To view overlaps and uniqueness of abundant and rare microbial species among oral health, UpSet plots were generated. The healthy group displayed the highest species count (93), followed by early periodontitis (75), while advanced periodontitis and diabetic periodontitis showed 74 each (Figure 3A). A core set of 44 species was shared among all groups. Further, 9 unique species from genera including *Streptococcus, Actinomyces Lactobacillus* were observed in healthy dental subgingival plaque samples. Among these, 7 unique species observed in early periodontitis included species from genera like *Bifidobacterium, Lactobacillus, Olsenella* (Supplementary table 2). There were 2 unique species pertaining to genera *Mogibacterium* and *Neisseria* found in advanced periodontitis, while only one unique species of *Capnocytophaga* was observed in diabetic periodontitis. Among the shared bacteria, 14 species were abundant in healthy, advanced periodontitis and diabetic periodontitis (Supplementary table 2). Additionally, intersections showed only limited shared species between diabetic periodontitis and advanced periodontitis as well as healthy and early periodontitis (Figure 3A) indicating a bacterial shift. Overall, the UpSet plot confirms the presence of a core oral microbiome and the increasingly divergent microbial sets with disease severity. For rare species (relative abundance of < 0.1% in each group), a total of 1,680 rare species were shared by all four subgingival sample groups (Figure 3B). Early periodontitis exhibited the largest set of unique rare species (193), followed by diabetic periodontitis (114). Nearly, 533 species shared amongst healthy, early periodontitis, and advanced periodontitis were found absent in diabetic periodontitis, indicating a potential loss of diversity induced by systemic diabetic conditions. Conversely, 220 species were shared exclusively between advanced periodontitis and diabetic periodontitis, potentially representing rare opportunists favored in the late-disease state. Smaller, unique intersections such as healthy and early periodontitis (92 species); healthy and advanced periodontitis (65 species) reflect transitional stages of microbial drift (Figure 3B). In contrast, only few species were found across rare combinations such as diabetic periodontitis and healthy, early periodontitis and advanced periodontitis (Figure 3B).

### 3.5 Metagenome based metabolic profiling

**Figure 4:**
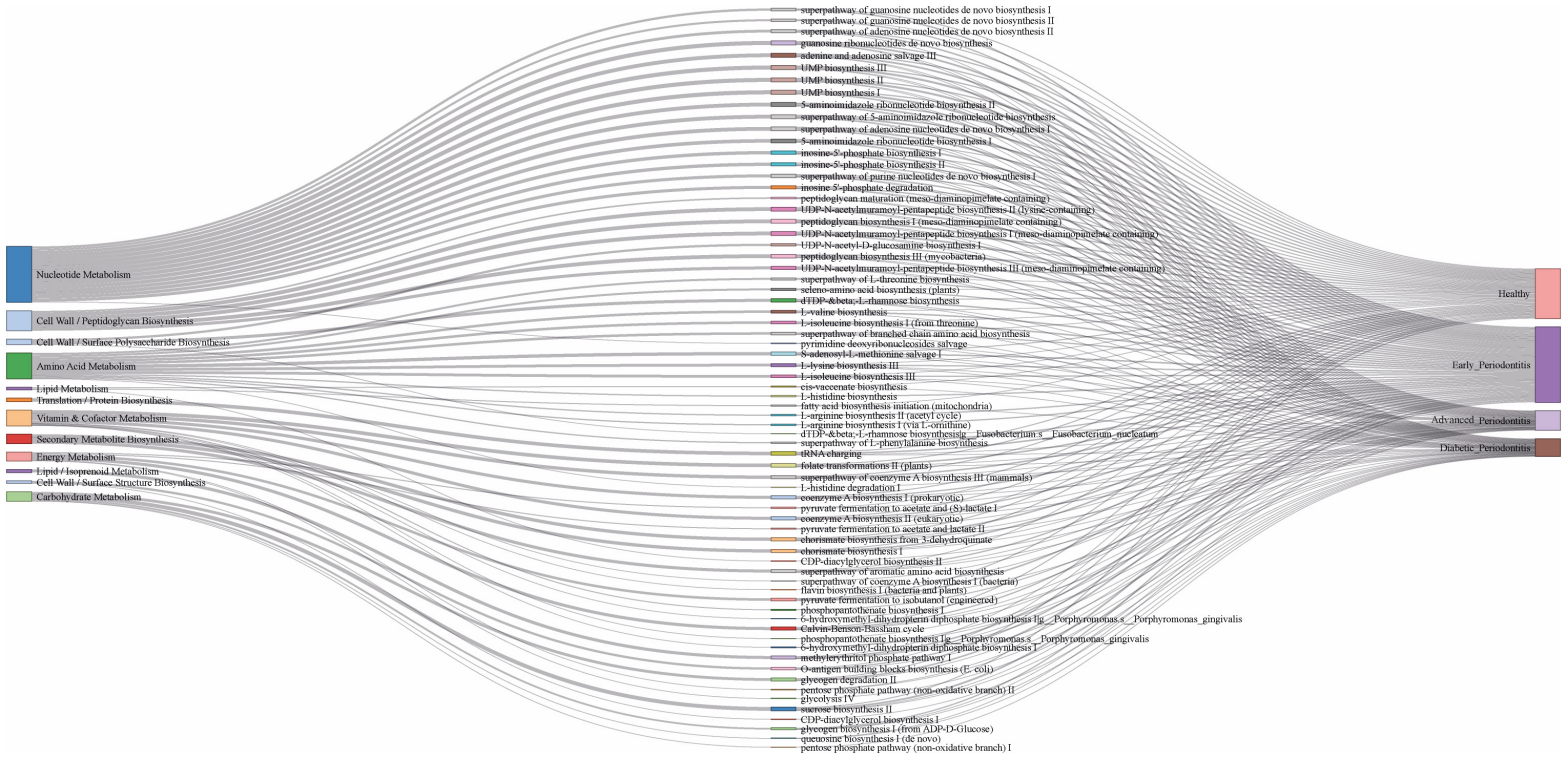
Sankey diagram depicts the comparative distribution of the 50 most abundant metabolic pathways identified among the four clinical groups including healthy, early periodontitis, advanced periodontitis, and diabetic periodontitis.

The comparative analysis of metabolic pathways reads per kilobases (RPKs) revealed a distinct abundance pattern across the four clinical categories (Supplementary Figure S1). Healthy subgingival reads showed enrichment in nucleotide biosynthesis (*de novo* biosynthesis, adenine and adenosine salvage III, and UMP biosynthesis I, II, and III; and peptidoglycan biosynthesis, consistent with core metabolic maintenance. However, early periodontitis displayed the highest overall pathways abundance with higher abundance for UMP biosynthesis I–III, guanosine ribonucleotides *de novo* biosynthesis, and sucrose biosynthesis II, alongside elevated amino acid metabolism in all categories. Advanced periodontitis sample's reads exhibited reduced abundances of UMP biosynthesis I–III, with vitamin/cofactor metabolism expanding to cell wall biosynthesis and nucleotide metabolism. Further, dTDP-β-L-rhamnose biosynthesis shows difference in RPKs between early periodontitis and advanced periodontitis (Supplementary Figure S1). Early periodontitis subgingival samples displayed unique amino acid biosynthesis pathways (L-arginine biosynthesis I & II, L-histidine biosynthesis, L-phenylalanine biosynthesis) among all categories. The advanced periodontitis subgingival samples showed unique degradative pathways (L-histidine degradation I) and stress-response vitamins (flavin biosynthesis I). On the other hand, diabetic periodontitis showed abundance of glycolysis metabolic pathways, tRNA splicing pathway and energy metabolism elevation. Interestingly, higher lipid metabolism was observed in diabetic periodontitis (Supplementary Figure S1). With all those pathways, diabetic sample's reads showed cell wall peptidoglycan biosynthesis and nucleotide pathways largely unchanged. Average pathway abundance revealed a metabolic efficiency collapse, like early periodontitis showed hyperactive metabolism, whereas healthy category showed a kind of balanced metabolism. However, during disease progression from early to advanced stage of periodontitis, a metabolic depression was observed. Diabetic periodontitis showed a similar metabolic shifts like advanced stage of periodontitis. Advanced periodontitis shows an increase in vitamin/cofactor pathways with respect to the healthy community, representing the highest abundance of any functional category. The Sankey diagram shows the comparative distribution of the top 50 most abundant metabolic pathways across the clinical groups (Figure 4). Healthy subgingival samples showed genes availability, particularly with pathways related to folate transformations, glycolysis, sucrose biosynthesis, and coenzyme A metabolism, while advanced periodontitis and diabetic periodontitis comparatively contained a lower gene number.

### 3.6 Pathobionts and probiotic microbiome analyses

**Figure 5:**
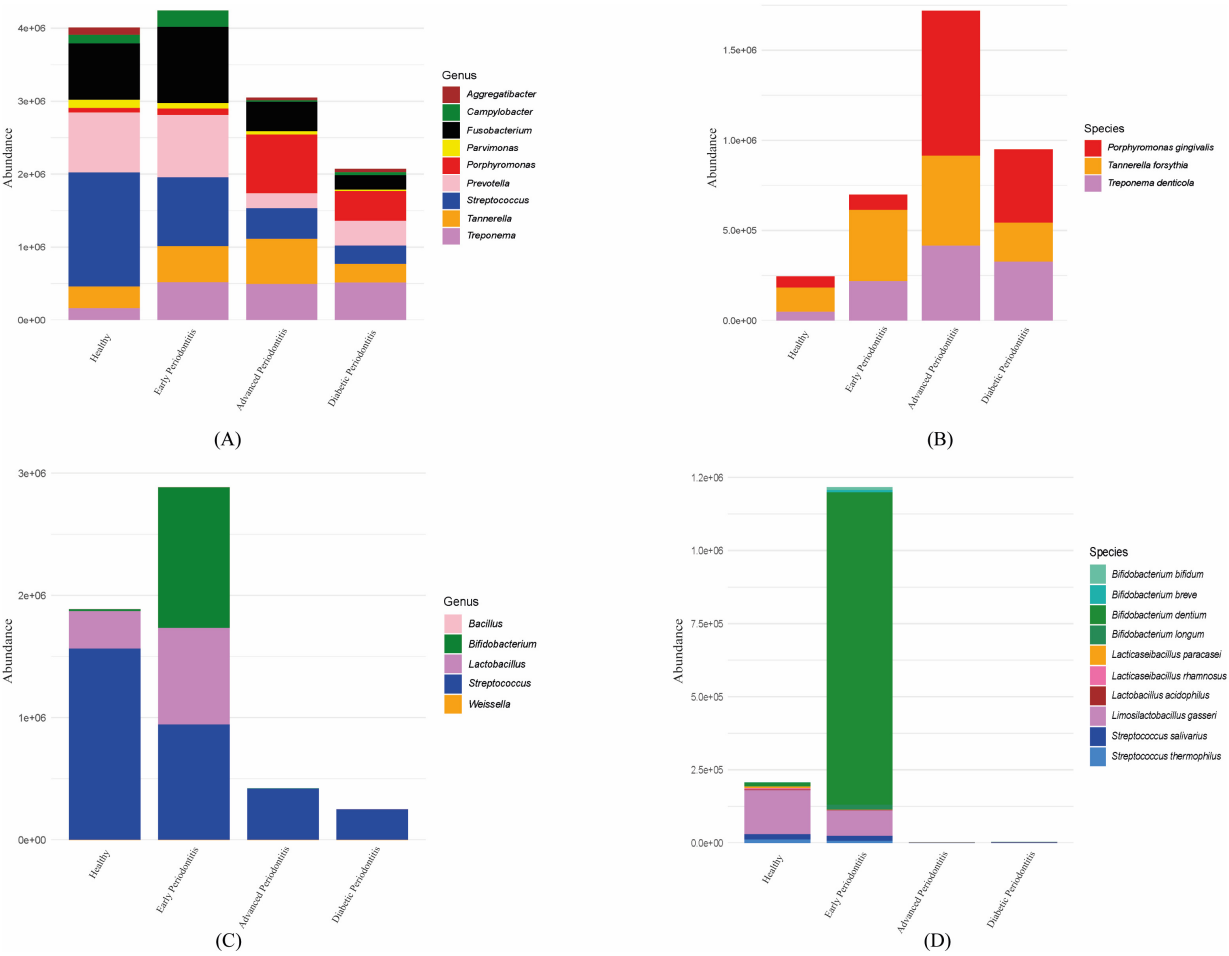
Genera and species level shifts in oral microbiome composition across periodontal health and disease groups. **(A)** Genera level stacked bar plot of total pathogen abundance. **(B)** Species-level bar plot of the Red Complex (*Porphyromonas gingivalis* in red, *Tannerella forsythia* in orange, *Treponema denticola* in magenta). **(C)** Stacked bar plot of total probiotics genera abundance across the four groups. **(D)** Stacked bar plot of species-level composition of key probiotic taxa.

Results of pathobionts showed expansion of typical periodontal pathogens, like *Porphyromonas, Tannerella* and *Treponema* genera (Figure 5A). Although pathogen diversity decreased in severe disease, the abundance of Red-complex species (*P. gingivalis, T. forsythia*, and *T. denticola*) specifically increased in advanced and diabetic periodontitis (Figure 5B). Probiotic profiles displayed biphasic changes in probiotic genera distribution (Figure 5C). Early periodontitis showed a significant increase in *Bifidobacterium* and *Lactobacillus* species (Figure 5D). In contrast, advanced and diabetic periodontitis showed loss of probiotic taxa, including near-complete depletion of *Lactobacillus* and also major reduction in *Streptococcus salivarius* and *S. thermophilus*.

## 4 Discussion

The metagenomic analysis of subgingival microbes across healthy, early periodontitis, advanced periodontitis and diabetic periodontitis provides evidence that both corroborates and challenges existing literature. Earlier studies have emphasized that the decline in microbial diversity is a common feature of periodontal disease progression (Abusleme et al., 2013). Our findings are in concordance with this ecological imbalance during the transition from healthy to early periodontitis. However, some recent reports have described an increased diversity in advanced periodontitis due to the influx of opportunistic colonizers (Abusleme et al., 2013). Contrary to this, data showed a reduced diversity in advanced periodontitis potentially reflecting differences in population background, sequencing depth or the resolution of shotgun metagenomics compared with 16S rRNA gene sequencing. Lower alpha diversity was observed in diseased compared to healthy groups. This shows that microbial load differs across periodontal disease stages, which is similar to the early dysbiosis model (Abusleme et al., 2013). Reduction of diversity in early periodontitis indicates early ecological disruptions and supports a dysbiosis-driven early stage of periodontitis. Distinct clustering in beta diversity plots and a greater dissimilarity in diabetic periodontitis reflect community reorganization influenced by host metabolism and disease stage. These results are consistent with the ecological plaque hypothesis and recent observations on hyperglycemia-driven dysbiosis (Zhao et al., 2023). Taxonomic shifts showed a tendency toward an anaerobic ecosystem with increasing periodontitis severity (Cugini et al., 2021). Further, enrichment of red-complex and reduction of commensals reflects dysbiosis trends (Shaikh et al., 2023). Increased occurrence of *Olsenella* and *B. dentium* in early-stage of periodontitis and pathogenic dominance in advanced stages, suggests stage-specific microbial profiles. These observations were consistent with early dysbiosis models (Siddiqui et al., 2023). Rare species expansion in early periodontitis and loss in diabetic periodontitis reflect niche opening followed by collapse under inflammatory or metabolic stress (Pascoal et al., 2025; Jousset et al., 2017). Loss of hundreds of species in diabetic periodontitis aligns with diabetes-driven microbial constriction (Lu et al., 2022). While the 101 abundant species showed dramatic shifts with different disease stages, the 3,289 rare species remain remarkably stable across all stages (Supplementary Table 2). This can give an estimation about a vast housekeeping microbiota that can provide metabolic resilience and genetic diversity. Functionally, the enrichment of carbohydrate metabolism in early stages of disease supports the ecological plaque hypothesis, which proposes that saccharolytic activity destabilizes the microbial community and primes it for a shift to dysbiosis (Lamont et al., 2018; Shi et al., 2020). In advanced disease stages, the dominance of amino acid and lipid metabolism observed is concordant with metabolomic studies demonstrating a proteolytic, inflammation adapted community in late periodontal cases (Li et al., 2023). In diabetic periodontitis, the further enrichment of virulence and stress response pathways supports a previous finding that hyperglycemia shapes microbial pathogenicity and inflammatory potential (Lu et al., 2022).

Stage-specific pathobiont analysis showed a considerable increase in red complex abundance with a reduction in commensal and opportunistic species. It showed a profile consistent with the severe inflammation-associated microbiomes described in literature (Hajishengallis, 2014). The observed biphasic probiotic response shows an initial compensatory probiotic microbiome followed by a reduction or decrease, suggests a potential therapeutic interface for microbial interventions (Krasse et al., 2006; Sachelarie et al., 2025). *Bifidobacterium* increase, especially *B. dentium*, highlights an opportunistic response under inflammatory stress conditions (Ventura et al., 2009). The influence of diabetes on periodontal microbial ecology has been a matter of consideration, with some studies confirming a minimal taxonomic difference between diabetic and non-diabetic disease (Casarin et al., 2013) whereas, other identified distinct microbiomes with higher pathogenic load (Shi et al., 2020). However, our finding is showing a clear divergence in diabetic periodontitis particularly the enrichment of red complex taxa, which is in accordance with the previous studies that showed diabetes significantly changes the microbial ecology of the periodontal environment (Lu et al., 2022). Earlier studies have identified taxonomically heterogeneous but functionally similar communities in chronic periodontitis (Wang et al., 2013; Huang et al., 2021) and have begun to elucidate the impact of diabetes on the subgingival microbiota and their functional potential (Shi et al., 2020; Lu et al., 2022). However, such analyses have generally compared healthy with a single chronic disease parameter without stratifying disease status by stage and metabolic phenotype. To our knowledge, this is the first shotgun metagenomic analysis to combine clinically staged early (Stage I/II) and advanced (Stage III/IV) periodontitis with diabetic periodontitis in a unified framework, albeit in a small exploratory dataset. This enabled us to hypothesize a stage-specific trajectory in which early disease is marked by reduced richness and probiotic enrichment, late disease by pathogen dominance and metabolic impairment, and diabetic periodontitis by further reprogramming of the microbiota toward glycolysis and lipid biosynthesis. Another aspect of study is the quantification of probiotic/pathobiont balance and rare biosphere structure at different stages of disease. By integrating a carefully assembled probiotics reference set with rare vs. abundant species classification, we observed a biphasic profile characterized by an early periodontitis stage dominated by health-associated genera like *Bifidobacterium* and *Lactobacillus*. In contrast, advanced and diabetic periodontitis stage exhibited depletion of these taxa and considerable loss of rare species from the shared gamma-diversity pool (Pascoal et al., 2025; Jousset et al., 2017). This profile is consistent with ecological theories in which an initial phase of compensatory expansion of beneficial commensals is overwhelmed by chronic inflammatory and metabolic stress (Lamont et al., 2018; Siddiqui et al., 2023).

By integrating a pooled-DNA shotgun metagenomics design, this study reveals that deep functional and taxonomic metagenomics analysis is possible even in resource-poor environments. Further, this approach lacks individual-level resolution and does not enable formal statistical inference, it offers a useful roadmap for exploratory metagenomic analysis (Teufel and Sobetzko, 2022). While this study limits the participant demographics to those aged between 26 and 52 years, the groups within this cohort were not specifically age-matched (Healthy: 26.8 ± 0.96; Early periodontitis: 35.0 ± 4.69; Advanced periodontitis: 46.0 ± 4.97; Diabetic periodontitis: 46.0 ± 5.23). However, this is an accurate representation of the natural progression of these diseases in the general population (Eke et al., 2012). Periodontitis is a cumulative process in which the rate of attachment loss accelerates with time, and the onset of diabetes in the general population (Eke et al., 2012; Centers for Disease Control and Prevention, 2026). However, as age is a confounding variable that inherently affects the oral microbiome, the slightly higher mean age in the advanced and diabetic groups will be a confounding variable in this study. The metagenomic libraries were prepared from DNA that was pooled at the group level as this approach increases the depth of sequencing and lowers costs, however, it is not possible to examine inter-individual variability (Ray et al., 2019). The alpha and beta diversity, taxonomic composition, and pathway abundance reported in this study must therefore, be considered as descriptive, hypothesis-generating group-level data. The cross-sectional nature of the study does not allow for the examination of temporal relationships or host immune and metabolic responses, and thus, there is a need for longitudinal studies with a multi-omics approach to test the hypotheses proposed in this study.

## 5 Conclusion

This study highlights the structural and functional shift of the subgingival microbiome across healthy, early and advanced periodontitis, and diabetic periodontitis using combined shotgun metagenomics. A healthy subgingival sample exhibited high microbial diversity and core metabolic pathways. Early-stage of disease (early periodontitis) was marked by hyper biosynthetic pathways response, with an increase in nucleotide and cell-wall pathway activity, and rise in *Bifidobacterium dentium*. Advanced periodontitis showed a collapse in dTDP-β-L-rhamnose biosynthesis alongside a surge in red complex pathogens. Diabetic periodontitis-maintained glycolysis and peptidoglycan biosynthesis under hyperglycemic conditions, with a considerable increase in lipid metabolism pathways. These results provide quantitative, stage associated microbial and metabolic markers for underscoring the importance of targeting early metabolic shifts to prevent dysbiosis. However, this hypothesis requires validation through larger, individual level prospective studies.

## Data Availability

The datasets presented in this study can be found in online repositories. The names of the repository/repositories and accession number(s) can be found in the article/Supplementary material.
