# Supplementary material for: Metagenomic insights into oral microbiota dynamics in diabetic and non-diabetic periodontal disease: a pilot study
Source: Front Microbiol. 2026 Apr 14;17:1799124. doi: 10.3389/fmicb.2026.1799124 (PMC13121355; doi:10.3389/fmicb.2026.1799124)
Supplement: Supplementary file 1 [file Data_Sheet_1.doc]

**Metagenomic Insights into Oral Microbiota Dynamics in Diabetic and Non-Diabetic Periodontal Disease: A Pilot Study**

Harshvardhan^1,2^, Mahaldeep Kaur^1,3^, Vishakha Grover^4^, Anil Kumar Pinnaka^1,2‡^, Suresh Korpole^1,2*^

^1^Microbial Type Culture Collection and Gene bank, CSIR-Institute of Microbial Technology, Sector 39A, Chandigarh 160036, India

^2^Academy of Scientific and Innovative Research (AcSIR), Ghaziabad 201002, India;

^3^Present address: Laboratory of Clinical Immunology and Microbiology, Epithelial Therapeutics Unit, National Institute of Allergy and Infectious Disease, National Institutes of Health, Bethesda, MD 20892, USA

^4^Dr. Harvansh Singh Judge Institute of Dental Sciences and Hospital, Panjab University, Chandigarh 160014, India

‡ **Co-correspondence:**Dr. Anil Kumar Pinnaka
[anilkumar.pinnaka@csir.res.in](mailto:anilkumar.pinnaka@csir.res.in)

*** Correspondence:**Dr. Suresh Korpole
[suresh.korpole@csir.res.in](mailto:suresh.korpole@csir.res.in)

Running title: Metagenomic insights into periodontitis

Supplementary table 1: Clinical and demographic characteristics of study participants across periodontal health and disease groups

| **Sample ID** | **Sequencing ID** | **Clinical group** | **Age** | **Sex** | **BMI** | **Smoking Status** | **Alcohol Use** | **Diabetes Status** | **Hypertension** | **Recent Antibiotic Use** | **BOP Score** | **PPD (mm)** | **CAL (mm)** | **Caries** | **Missing Teeth** | **DNA Pooling** |
| --- | --- | --- | --- | --- | --- | --- | --- | --- | --- | --- | --- | --- | --- | --- | --- | --- |
| 11 | DH2 | Healthy | 26 | F | 20 | No | No | No | No | No | 10% | 1 | 0 | No | 0 | Yes |
| 12 |  | Healthy | 28 | F | 19 | No | No | No | No | No | 12% | 2 | 0 | No | 0 |  |
| 13 |  | Healthy | 27 | F | 21 | No | No | No | No | No | 15% | 2 | 0 | No | 0 |  |
| 14 |  | Healthy | 26 | F | 23 | No | No | No | No | No | 10% | 2 | 0 | No | 0 |  |
| 1 | DP1 | Stage I/II periodontitis | 34 | F | 21 | No | No | No | No | No | 20% | 2 | 3 | No | 0 | Yes |
| 2 |  | Stage I/II periodontitis | 40 | M | 28 | No | No | No | No | No | 25% | 3 | 3 | No | 0 |  |
| 4 |  | Stage I/II periodontitis | 29 | M | 26 | No | No | No | No | No | 30% | 4 | 4 | No | 0 |  |
| 5 |  | Stage I/II periodontitis | 37 | F | 28 | No | No | No | No | No | 45% | 4 | 4 | No | 0 |  |
| 6 | DP2 | Stage III/IVperiodontitis | 39 | M | 30 | No | No | No | No | No | 60% | 7 | 9 | No | 2 | Yes |
| 7 |  | Stage III/IVperiodontitis | 48 | M | 34 | No | No | No | No | No | 70% | 10 | 8 | No | 6 |  |
| 8 | DP4 | Stage III/IVperiodontitis | 50 | F | 28 | No | No | No | No | No | 55% | 8 | 5 | No | 6 | Yes |
| 10 |  | Stage III/IVperiodontitis | 47 | M | 32 | No | No | No | No | No | 62% | 8 | 6 | No | 5 |  |
| 3 | DPD | Diabetic Periodontitis | 42 | M | 30 | No | No | Hb A1C-7 | No | No | 60% | 8 | 6 | No | 0 | Yes |
| 17 |  | Diabetic Periodontitis | 41 | F | 28 | No | No | Hb A1C-6.5 | No | No | 50% | 7 | 9 | No | 4 |  |
| 18 |  | Diabetic Periodontitis | 52 | F | 24 | No | No | Hb A1C-8 | No | No | 65% | 10 | 8 | No | 6 |  |
| 19 |  | Diabetic Periodontitis | 49 | M | 28 | No | No | Hb A1C-11 | No | No | 66% | 8 | 11 | No | 7 |  |

Supplementary table 2: Shared and group-restricted taxa across clinical categories. A comparative table of abundant/shared species (total 101) and rare/unique species (Total 3349) across all four clinical groups. Species lists are isolated during UpSet plot species-level profiling. Here, species observed as abundant across the indicated groups (left) versus species uniquely/rarely detected in those groups relative to others (right), as defined in the analysis workflow. Count shows the number of species listed in that cell.

|  | **Abundant Taxa** | | **Rare Taxa** | |
| --- | --- | --- | --- | --- |
| **Category** | **Abundant/Shared Species** | **Count** | **Rare/Unique Species** | **Count** |
| Healthy + Early_Periodontitis + Advanced_Periodontitis + Diabetic_Periodontitis | *Actinomyces oris, Actinomyces radicidentis, Actinomyces sp. Z16, Actinomyces sp. oral taxon 414, Actinomyces sp. oral taxon 897, Campylobacter gracilis, Capnocytophaga gingivalis, Capnocytophaga leadbetteri, Capnocytophaga sp. ChDC OS43, Capnocytophaga sp. oral taxon 323, Capnocytophaga sputigena, Fusobacterium hwasookii, Fusobacterium nucleatum, Fusobacterium periodonticum, Leptotrichia buccalis, Leptotrichia sp. oral taxon 498, Leptotrichia sp. oral taxon 847, Olsenella sp. oral taxon 807, Ottowia sp. oral taxon 894, Parvimonas micra, Porphyromonas gingivalis, Prevotella denticola, Prevotella enoeca, Prevotella intermedia, Prevotella melaninogenica, Pseudopropionibacterium propionicum, Selenomonas sp. oral taxon 126, Selenomonas sp. oral taxon 136, Selenomonas sp. oral taxon 478, Selenomonas sp. oral taxon 920, Selenomonas sputigena, Streptococcus anginosus, Streptococcus gordonii, Streptococcus intermedius Streptococcus mitis, Streptococcus oralis, Streptococcus pneumoniae, Streptococcus sp. NPS 308, Streptococcus sp. oral taxon 064 Tannerella forsythia, Tannerella sp. oral taxon HOT-286, Treponema denticola, Treponema sp. OMZ 838, Veillonella parvula* | 44 | *Acaryochloris marina, Acetoanaerobium sticklandii, Acetobacter aceti, Acetobacter ghanensis, Acetobacter oryzifermentans, Acetobacter pasteurianus, Acetobacter persici, Acetobacter senegalensis, Acetobacterium sp. KB-1, Acetobacterium woodii, Acetohalobium arabaticum, Acetomicrobium mobile, Acholeplasma brassicae, Acholeplasma laidlawii, Acholeplasma oculi, Acholeplasma palmae, Achromobacter denitrificans, Achromobacter insolitus, Achromobacter sp. AONIH1, Achromobacter sp. B7, Achromobacter sp. MFA1 R4, Achromobacter spanius, Achromobacter xylosoxidans, Acidaminococcus fermentans, Acidaminococcus intestini, Acidianus brierleyi, Acidiferrobacter sp. SPIII_3, Acidihalobacter ferrooxidans, Acidihalobacter prosperus, Acidilobus sp. 7A, Acidimicrobium ferrooxidans, Acidipropionibacterium acidipropionici, Acidipropionibacterium virtanenii, Acidisphaera sp. G45-3, Acidithiobacillus caldus, Acidithiobacillus ferrivorans, Acidithiobacillus ferrooxidans, Acidobacteriaceae bacterium SBC82, Acidobacterium capsulatum, Acidothermus cellulolyticus, Acidovorax avenae, Acidovorax carolinensis, Acidovorax cattleyae, Acidovorax citrulli, Acidovorax ebreus, Acidovorax sp. JS42, Acidovorax sp. KKS102, Acidovorax sp. RAC01, Acidovorax sp. T1, Aciduliprofundum sp. MAR08-339, Acinetobacter baumannii, Acinetobacter calcoaceticus, Acinetobacter defluvii, Acinetobacter equi, Acinetobacter haemolyticus, Acinetobacter indicus, Acinetobacter junii, Acinetobacter lactucae, Acinetobacter larvae, Acinetobacter lwoffii, Acinetobacter nosocomialis, Acinetobacter oleivorans, Acinetobacter pittii, Acinetobacter radioresistens, Acinetobacter schindleri, Acinetobacter soli, Acinetobacter sp. ACNIH1, Acinetobacter sp. ACNIH2, Acinetobacter sp. ADP1, Acinetobacter sp. NCu2D-2, Acinetobacter sp. SWBY1, Acinetobacter sp. TGL-Y2, Acinetobacter sp. TTH0-4, Acinetobacter sp. WCHA55, Acinetobacter sp. WCHAc010005, Acinetobacter sp. WCHAc010034, Acinetobacter venetianus, Acinetobacter wuhouensis, Actinoalloteichus hoggarensis, Actinoalloteichus hymeniacidonis, Actinoalloteichus sp. ADI127-7, Actinoalloteichus sp. AHMU CJ021, Actinobacillus equuli, Actinobacillus pleuropneumoniae, Actinobacillus porcitonsillarum, Actinobacillus succinogenes, Actinobacillus suis, Actinobacteria bacterium IMCC19121, Actinobacteria bacterium IMCC25003, Actinobacteria bacterium IMCC26103, Actinobacteria bacterium IMCC26256, Actinomadura amylolytica, Actinomyces pacaensis, Actinomyces radingae, Actinomyces sp. VUL4_3, Actinoplanes derwentensis, Actinoplanes friuliensis, Actinoplanes missouriensis, Actinoplanes sp. N902-109, Actinopolymorpha singaporensis, Actinopolyspora erythraea, Actinosynnema mirum, Actinosynnema pretiosum, Actinotignum schaalii, Actinotignum sp. 313, Adlercreutzia equolifaciens, Advenella kashmirensis, Advenella mimigardefordensis, Aequorivita sublithincola, Aeribacillus pallidus, Aerococcaceae bacterium ZY16052, Aerococcus christensenii, Aerococcus sanguinicola, Aerococcus urinae, Aerococcus urinaeequi, Aerococcus urinaehominis, Aerococcus viridans, Aeromicrobium choanae, Aeromicrobium erythreum, Aeromicrobium marinum, Aeromicrobium sp. 592, Aeromicrobium sp. A1-2, Aeromonas hydrophila, Aeromonas media, Aeromonas phage CC2, Aeromonas rivipollensis, Aeromonas salmonicida, Aeromonas schubertii, Aeromonas sp. ASNIH3, Aeromonas sp. ASNIH4, Aeromonas sp. CA23, Aeromonas sp. CU5, Aeromonas veronii, Afipia sp. GAS231, Agarilytica rhodophyticola, Agarivorans gilvus, Aggregatibacter actinomycetemcomitans, Agrobacterium fabrum, Agrobacterium rhizogenes, Agrobacterium sp. H13-3, Agrobacterium sp. RAC06, Agrobacterium tumefaciens, Agrobacterium vitis, Agrococcus carbonis, Agrococcus jejuensis, Agromyces aureus, Agromyces flavus, Agromyces sp. 30A, Ahniella affigens, Akkermansia glycaniphila, Akkermansia muciniphila, Alcaligenes faecalis, Alcanivorax dieselolei, Alcanivorax pacificus, Alcanivorax sp. N3-2A, Alcanivorax xenomutans, Algibacter alginicilyticus, Algoriphagus machipongonensis, Algoriphagus sp. M8-2, Alicycliphilus denitrificans, Alicyclobacillus acidocaldarius, Aliiarcobacter butzleri, Aliiarcobacter skirrowii, Aliiarcobacter trophiarum, Aliivibrio fischeri, Aliivibrio salmonicida, Aliivibrio wodanis, Alistipes finegoldii, Alistipes shahii, Alkalilimnicola ehrlichii, Alkaliphilus metalliredigens, Alkaliphilus oremlandii, Alkalitalea saponilacus, Alloactinosynnema sp. L-07, Allochromatium vinosum, Allokutzneria albata, Altererythrobacter atlanticus, Altererythrobacter dongtanensis, Altererythrobacter epoxidivorans, Altererythrobacter ishigakiensis, Altererythrobacter mangrovi, Altererythrobacter marensis, Altererythrobacter namhicola, Altererythrobacter sp. B11, Altererythrobacter sp. ZODW24, Alteromonas australica, Alteromonas macleodii, Alteromonas mediterranea, Alteromonas naphthalenivorans, Alteromonas sp. BL110, Alteromonas sp. RKMC-009, Aminobacter aminovorans, Aminobacter sp. MSH1, Aminobacterium colombiense, Aminomonas paucivorans, Ammonifex degensii, Amphibacillus xylanus, Amycolatopsis albispora, Amycolatopsis japonica, Amycolatopsis keratiniphila, Amycolatopsis mediterranei, Amycolatopsis methanolica, Amycolatopsis orientalis, Amycolatopsis sp. AA4, Amycolatopsis sp. BJA-103, Anabaena cylindrica, Anabaena sp. WA102, Anaerococcus mediterraneensis, Anaerococcus prevotii, Anaerolinea thermophila, Anaeromyxobacter dehalogenans, Anaeromyxobacter sp. Fw109-5, Anaeromyxobacter sp. K, Anaerostipes hadrus, Anaerotignum propionicum, Anaplasma phagocytophilum, Anderseniella sp. Alg231-50, Aneurinibacillus soli, Aneurinibacillus sp. XH2, Anoxybacillus amylolyticus, Anoxybacillus flavithermus, Antarctobacter heliothermus, Aquabacterium olei, Aquaspirillum sp. LM1, Aquiflexum balticum, Aquimarina sp. AD1, Aquimarina sp. AD10, Aquimarina sp. BL5, Aquitalea magnusonii, Arachidicoccus sp. BS20, Arachidicoccus sp. KIS59-12, Arcanobacterium haemolyticum, Arcanobacterium phocae, Archaeoglobus veneficus, Archangium gephyra, Arcobacter nitrofigilis, Arcobacter sp. L, Arcobacter sp. LPB0137, Arcticibacterium luteifluviistationis, Arenibacter algicola, Aromatoleum aromaticum, Arsenicicoccus sp. oral taxon 190, Arthrobacter alpinus, Arthrobacter crystallopoietes, Arthrobacter sp. ATCC 21022, Arthrobacter sp. DCT5, Arthrobacter sp. ERGS1:01, Arthrobacter sp. FB24, Arthrobacter sp. PAMC 25486, Arthrobacter sp. PGP41, Arthrobacter sp. QXT-31, Arthrobacter sp. Rue61a, Arthrobacter sp. U41, Arthrobacter sp. YC-RL1, Arthrobacter sp. YN, Arthrobacter sp. ZXY-2, Arthrospira platensis, Asaia bogorensis, Asticcacaulis excentricus, Aurantimicrobium minutum, Aurantimicrobium sp. MWH-Mo1, Aurantimicrobium sp. MWH-Uga1, Auraticoccus monumenti, Aureimonas sp. AU20, Aureitalea sp. RR4-38, Auricoccus indicus, Auritidibacter sp. NML130574, Austwickia chelonae, Azoarcus communis, Azoarcus olearius, Azoarcus sp. CIB, Azoarcus sp. KH32C, Azoarcus sp. SY39, Azorhizobium caulinodans, Azospira oryzae, Azospirillum brasilense, Azospirillum humicireducens, Azospirillum lipoferum, Azospirillum sp. M2T2B2, Azospirillum sp. TSH58, Azospirillum thiophilum, Azotobacter chroococcum, Azotobacter vinelandii, Bacillus altitudinis, Bacillus amyloliquefaciens, Bacillus anthracis, Bacillus atrophaeus, Bacillus beveridgei, Bacillus butanolivorans, Bacillus cellulosilyticus, Bacillus cereus, Bacillus clausii, Bacillus coagulans, Bacillus cohnii, Bacillus cytotoxicus, Bacillus filamentosus, Bacillus flexus, Bacillus glycinifermentans, Bacillus gobiensis, Bacillus halodurans, Bacillus horikoshii, Bacillus infantis, Bacillus jeotgali, Bacillus kochii, Bacillus krulwichiae, Bacillus lehensis, Bacillus lentus, Bacillus megaterium, Bacillus methanolicus, Bacillus muralis, Bacillus mycoides, Bacillus oceanisediminis, Bacillus paralicheniformis, Bacillus pseudofirmus, Bacillus pseudomycoides, Bacillus pumilus, Bacillus safensis, Bacillus simplex, Bacillus smithii, Bacillus sonorensis, Bacillus sp. 1NLA3E, Bacillus sp. FJAT-18017, Bacillus sp. FJAT-22090, Bacillus sp. FJAT-45348, Bacillus sp. MD-5, Bacillus sp. OxB-1, Bacillus sp. WP8, Bacillus sp. X1(2014), Bacillus sp. Y-01, Bacillus sp. Y1, Bacillus subtilis, Bacillus thermoamylovorans, Bacillus thuringiensis, Bacillus velezensis, Bacillus weihaiensis, Bacillus xiamenensis, Bacterioplanes sanyensis, Bacteriovorax stolpii, Bacteroidales bacterium CF, Bacteroides caccae, Bacteroides caecimuris, Bacteroides cellulosilyticus, Bacteroides coprosuis, Bacteroides dorei, Bacteroides fragilis, Bacteroides helcogenes, Bacteroides heparinolyticus, Bacteroides ovatus, Bacteroides salanitronis, Bacteroides thetaiotaomicron, Bacteroides vulgatus, Bacteroides zoogleoformans, Bacteroidetes bacterium Omega, Barnesiella viscericola, Bartonella ancashensis, Bartonella apis, Bartonella australis, Bartonella bovis, Bartonella schoenbuchensis, Bartonella sp. 1-1C, Bartonella sp. WD16.2, Bartonella vinsonii, Basilea psittacipulmonis, Bathymodiolus septemdierum thioautotrophic gill symbiont, Bdellovibrio bacteriovorus, Bdellovibrio exovorus, Beggiatoa leptomitoformis, Beijerinckia indica, Belliella baltica, Bernardetia litoralis, Betaproteobacteria bacterium GR16-43, Beutenbergia cavernae, Bibersteinia trehalosi, Bifidobacterium actinocoloniiforme, Bifidobacterium adolescentis, Bifidobacterium angulatum, Bifidobacterium animalis, Bifidobacterium asteroides, Bifidobacterium bifidum, Bifidobacterium breve, Bifidobacterium choerinum, Bifidobacterium coryneforme, Bifidobacterium indicum, Bifidobacterium kashiwanohense, Bifidobacterium pseudocatenulatum, Bifidobacterium pseudolongum, Bifidobacterium scardovii, Bifidobacterium thermophilum, Blastochloris viridis, Blastococcus saxobsidens, Blattabacterium clevelandi, Blattabacterium cuenoti, Blattabacterium punctulatus, Blattabacterium sp. (Mastotermes darwiniensis), Blattabacterium sp. (Nauphoeta cinerea), Blautia hansenii, Blautia sp. N6H1-15, Blochmannia endosymbiont of Camponotus (Colobopsis) obliquus, Blochmannia endosymbiont of Polyrhachis (Hedomyrma) turneri, Bordetella avium, Bordetella bronchialis, Bordetella bronchiseptica, Bordetella flabilis, Bordetella genomosp. 13, Bordetella genomosp. 8, Bordetella genomosp. 9, Bordetella hinzii, Bordetella holmesii, Bordetella parapertussis, Bordetella petrii, Bordetella pseudohinzii, Bordetella sp. H567, Bordetella sp. HZ20, Bordetella sp. N, Bordetella trematum, Borrelia crocidurae, Borrelia turcica, Borreliella valaisiana, Bosea sp. AS-1, Bosea sp. PAMC 26642, Bosea sp. RAC05, Bosea vaviloviae, Brachybacterium faecium, Brachybacterium ginsengisoli, Brachybacterium saurashtrense, Brachybacterium sp. P6-10-X1, Brachybacterium sp. VM2412, Brachybacterium sp. VR2415, Brachyspira hampsonii, Brachyspira hyodysenteriae, Brachyspira intermedia, Brachyspira murdochii, Brachyspira pilosicoli, Bradymonas sediminis, Bradyrhizobiaceae bacterium SG-6C, Bradyrhizobium diazoefficiens, Bradyrhizobium erythrophlei, Bradyrhizobium icense, Bradyrhizobium japonicum, Bradyrhizobium lablabi, Bradyrhizobium oligotrophicum, Bradyrhizobium ottawaense, Bradyrhizobium sp. 2 39S1MB, Bradyrhizobium sp. 3 85S1MB, Bradyrhizobium sp. BTAi1, Bradyrhizobium sp. CCGE-LA001, Bradyrhizobium sp. ORS 278, Bradyrhizobium sp. ORS 285, Bradyrhizobium sp. S23321, Bradyrhizobium sp. SK17, Bradyrhizobium sp. WSM471, Brenneria goodwinii, Brenneria sp. EniD312, Breoghania sp. L-A4, Brevefilum fermentans, Brevibacillus brevis, Brevibacillus formosus, Brevibacillus laterosporus, Brevibacterium linens, Brevibacterium sandarakinum, Brevibacterium siliguriense, Brevirhabdus pacifica, Brevundimonas diminuta, Brevundimonas naejangsanensis, Brevundimonas sp. DS20, Brevundimonas sp. GW460-12-10-14-LB2, Brevundimonas sp. LM2, Brevundimonas subvibrioides, Brevundimonas vesicularis, Brochothrix thermosphacta, Buchnera aphidicola, Burkholderia ambifaria, Burkholderia anthina, Burkholderia cenocepacia, Burkholderia cepacia, Burkholderia contaminans, Burkholderia diffusa, Burkholderia gladioli, Burkholderia glumae, Burkholderia insecticola, Burkholderia lata, Burkholderia metallica, Burkholderia multivorans, Burkholderia oklahomensis, Burkholderia plantarii, Burkholderia pseudomallei, Burkholderia pyrrocinia, Burkholderia seminalis, Burkholderia sp. BDU6, Burkholderia sp. BDU8, Burkholderia sp. Bp7605, Burkholderia sp. CCGE1001, Burkholderia sp. CCGE1002, Burkholderia sp. CCGE1003, Burkholderia sp. HB1, Burkholderia sp. IDO3, Burkholderia sp. JP2-270, Burkholderia sp. KK1, Burkholderia sp. LA-2-3-30-S1-D2, Burkholderia sp. MSMB0856, Burkholderia sp. OLGA172, Burkholderia sp. PAMC 26561, Burkholderia sp. PAMC 28687, Burkholderia sp. RPE67, Burkholderia sp. YI23, Burkholderia stabilis, Burkholderia stagnalis, Burkholderia territorii, Burkholderia thailandensis, Burkholderia ubonensis, Burkholderia vietnamiensis, Burkholderiales bacterium GJ-E10, Burkholderiales bacterium JOSHI_001, Burkholderiales bacterium YL45, Butyrivibrio fibrisolvens, Butyrivibrio hungatei, Butyrivibrio proteoclasticus, Caldanaerobacter subterraneus, Caldicellulosiruptor hydrothermalis, Caldicellulosiruptor owensensis, Caldicellulosiruptor saccharolyticus, Caldilinea aerophila, Caldimicrobium thiodismutans, Caldisericum exile, Calditerrivibrio nitroreducens, Caldithrix abyssi, Calothrix brevissima, Calothrix parasitica, Calothrix parietina, Calothrix sp. 336/3, Calothrix sp. NIES-2098, Calothrix sp. NIES-2100, Calothrix sp. NIES-3974, Calothrix sp. NIES-4101, Calothrix sp. PCC 7507, Calyptogena okutanii thioautotrophic gill symbiont, Campylobacter avium, Campylobacter coli, Campylobacter cuniculorum, Campylobacter curvus, Campylobacter fetus, Campylobacter helveticus, Campylobacter hepaticus, Campylobacter hominis, Campylobacter hyointestinalis, Campylobacter iguaniorum, Campylobacter insulaenigrae, Campylobacter jejuni, Campylobacter lanienae, Campylobacter lari, Campylobacter peloridis, Campylobacter pinnipediorum, Campylobacter sp. NCTC 13003, Campylobacter sp. RM12175, Campylobacter sp. RM6137, Campylobacter sp. RM8964, Campylobacter sputorum, Campylobacter subantarcticus, Campylobacter ureolyticus, Campylobacter volucris, Candidatus Accumulibacter phosphatis, Candidatus Amoebophilus asiaticus, Candidatus Aquiluna sp. UB-MaderosW2red, Candidatus Arsenophonus lipoptenae, Candidatus Arthromitus sp. SFB-mouse, Candidatus Arthromitus sp. SFB-rat-Yit, Candidatus Atelocyanobacterium thalassa, Candidatus Azobacteroides pseudotrichonymphae, Candidatus Babela massiliensis, Candidatus Baumannia cicadellinicola, Candidatus Cardinium hertigii, Candidatus Cloacimonas acidaminovorans, Candidatus Desulfofervidus auxilii, Candidatus Desulforudis audaxviator, Candidatus Desulfovibrio trichonymphae, Candidatus Endolissoclinum faulkneri, Candidatus Enterovibrio luxaltus, Candidatus Filomicrobium marinum, Candidatus Fokinia solitaria, Candidatus Fukatsuia symbiotica, Candidatus Gracilibacteria bacterium HOT-871, Candidatus Hamiltonella defensa, Candidatus Hoaglandella endobia, Candidatus Izimaplasma sp. HR1, Candidatus Kinetoplastibacterium crithidii, Candidatus Kinetoplastibacterium desouzaii, Candidatus Kinetoplastibacterium oncopeltii, Candidatus Koribacter versatilis, Candidatus Kuenenia stuttgartiensis, Candidatus Liberibacter africanus, Candidatus Methanomassiliicoccus intestinalis, Candidatus Methanomethylophilus alvus, Candidatus Methanoplasma termitum, Candidatus Methylopumilus turicensis, Candidatus Midichloria mitochondrii, Candidatus Moranella endobia, Candidatus Nanopelagicus abundans, Candidatus Nanopelagicus hibericus, Candidatus Nitrosoglobus terrae, Candidatus Nitrospira inopinata, Candidatus Pelagibacter sp. HIMB1321, Candidatus Pelagibacter sp. IMCC9063, Candidatus Pelagibacter sp. RS39, Candidatus Pelagibacter sp. RS40, Candidatus Pelagibacter ubique, Candidatus Phaeomarinobacter ectocarpi, Candidatus Phycorickettsia trachydisci, Candidatus Phytoplasma mali, Candidatus Planktophila dulcis, Candidatus Planktophila lacus, Candidatus Planktophila limnetica, Candidatus Planktophila sulfonica, Candidatus Planktophila vernalis, Candidatus Planktophila versatilis, Candidatus Promineofilum breve, Candidatus Protochlamydia amoebophila, Candidatus Protochlamydia naegleriophila, Candidatus Puniceispirillum marinum, Candidatus Rhodoluna planktonica, Candidatus Riesia pediculischaeffi, Candidatus Ruthia magnifica, Candidatus Saccharibacteria bacterium YM_S32_TM7_50_20, Candidatus Sodalis pierantonius, Candidatus Solibacter usitatus, Candidatus Sulcia muelleri, Candidatus Symbiobacter mobilis, Candidatus Tachikawaea gelatinosa, Candidatus Thiodictyon syntrophicum, Candidatus Thioglobus autotrophicus, Candidatus Thioglobus singularis, Capnocytophaga canimorsus, Capnocytophaga cynodegmi, Capnocytophaga haemolytica, Capnocytophaga sp. H2931, Capnocytophaga sp. H4358, Capnocytophaga stomatis, Carboxydocella thermautotrophica, Carboxydothermus hydrogenoformans, Carnobacterium divergens, Carnobacterium inhibens, Carnobacterium maltaromaticum, Carnobacterium sp. 17-4, Carnobacterium sp. CP1, Castellaniella defragrans, Catenovulum sp. CCB-QB4, Catenulispora acidiphila, Caulobacter henricii, Caulobacter mirabilis, Caulobacter segnis, Caulobacter sp. K31, Caulobacter vibrioides, Caulobacteraceae bacterium OTSz_A_272, Cedecea neteri, Celeribacter baekdonensis, Celeribacter ethanolicus, Celeribacter indicus, Celeribacter manganoxidans, Celeribacter marinus, Cellulomonas fimi, Cellulomonas flavigena, Cellulomonas gilvus, Cellulomonas sp. PSBB021, Cellulophaga algicola, Cellulophaga baltica, Cellulophaga lytica, Cellulosilyticum lentocellum, Cellulosimicrobium cellulans, Cellulosimicrobium sp. TH-20, Cellvibrio japonicus, Cellvibrio sp. PSBB006, Cellvibrio sp. PSBB023, Chamaesiphon minutus, Chania multitudinisentens, Chelativorans sp. BNC1, Chelatococcus daeguensis, Chelatococcus sp. CO-6, Chitinophaga caeni, Chitinophaga pinensis, Chitinophaga sp. MD30, Chlamydia felis, Chlamydia muridarum, Chlamydia pecorum, Chlamydia psittaci, Chlamydia sp. H15-1957-10C, Chlamydia suis, Chlamydia trachomatis, Chloracidobacterium thermophilum, Chlorobaculum limnaeum, Chlorobaculum parvum, Chlorobaculum tepidum, Chlorobium chlorochromatii, Chlorobium limicola, Chlorobium phaeobacteroides, Chlorobium phaeovibrioides, Chloroflexus aggregans, Chloroflexus aurantiacus, Chloroherpeton thalassium, Chondrocystis sp. NIES-4102, Chondromyces crocatus, Christensenella massiliensis, Chromatiaceae bacterium 2141T.STBD.0c.01a, Chromobacterium rhizoryzae, Chromobacterium sp. ATCC 53434, Chromobacterium sp. IIBBL 112-1, Chromobacterium sp. IIBBL 274-1, Chromobacterium vaccinii, Chromobacterium violaceum, Chromohalobacter salexigens, Chroococcidiopsis thermalis, Chryseobacterium camelliae, Chryseobacterium gallinarum, Chryseobacterium glaciei, Chryseobacterium indologenes, Chryseobacterium piperi, Chryseobacterium sp. IHB B 17019, Chryseobacterium sp. StRB126, Chryseobacterium sp. T16E-39, Chryseobacterium taklimakanense, Chryseolinea sp. KIS68-18, Chthonomonas calidirosea, Citrobacter amalonaticus, Citrobacter farmeri, Citrobacter freundii, Citrobacter koseri, Citrobacter rodentium, Citrobacter werkmanii, Citromicrobium sp. JL477, Clavibacter michiganensis, Cloacibacillus porcorum, Clostridiaceae bacterium 14S0207, Clostridiales bacterium CCNA10, Clostridioides difficile, Clostridium aceticum, Clostridium acetobutylicum, Clostridium argentinense, Clostridium autoethanogenum, Clostridium baratii, Clostridium beijerinckii, Clostridium bornimense, Clostridium botulinum, Clostridium butyricum, Clostridium carboxidivorans, Clostridium cellulovorans, Clostridium chauvoei, Clostridium cochlearium, Clostridium drakei, Clostridium estertheticum, Clostridium formicaceticum, Clostridium isatidis, Clostridium kluyveri, Clostridium ljungdahlii, Clostridium novyi, Clostridium pasteurianum, Clostridium perfringens, Clostridium saccharobutylicum, Clostridium saccharoperbutylacetonicum, Clostridium scatologenes, Clostridium septicum, Clostridium sp. BNL1100, Clostridium sp. DL-VIII, Clostridium sp. JN500901, Clostridium sp. MF28, Clostridium sp. SY8519, Clostridium sporogenes, Clostridium taeniosporum, Clostridium tetani, Clostridium tyrobutyricum, Cnuibacter physcomitrellae, Cobetia marina, Cohaesibacter sp. ES.047, Collimonas arenae, Collimonas fungivorans, Collimonas pratensis, Collinsella aerofaciens, Colwellia beringensis, Colwellia psychrerythraea, Colwellia sp. Arc7-D, Colwellia sp. MT41, Colwellia sp. PAMC 20917, Colwellia sp. PAMC 21821, Comamonadaceae bacterium A1, Comamonadaceae bacterium B1, Comamonas aquatica, Comamonas kerstersii, Comamonas serinivorans, Comamonas testosteroni, Conexibacter woesei, Congregibacter litoralis, Coprothermobacter proteolyticus, Coraliomargarita akajimensis, Corallococcus coralloides, Coriobacteriaceae bacterium 68-1-3, Coriobacterium glomerans, Corynebacterium ammoniagenes, Corynebacterium aquilae, Corynebacterium argentoratense, Corynebacterium atypicum, Corynebacterium aurimucosum, Corynebacterium callunae, Corynebacterium camporealensis, Corynebacterium casei, Corynebacterium crudilactis, Corynebacterium cystitidis, Corynebacterium deserti, Corynebacterium diphtheriae, Corynebacterium doosanense, Corynebacterium efficiens, Corynebacterium epidermidicanis, Corynebacterium falsenii, Corynebacterium flavescens, Corynebacterium frankenforstense, Corynebacterium genitalium, Corynebacterium glaucum, Corynebacterium glutamicum, Corynebacterium glyciniphilum, Corynebacterium halotolerans, Corynebacterium humireducens, Corynebacterium imitans, Corynebacterium jeikeium, Corynebacterium kroppenstedtii, Corynebacterium kutscheri, Corynebacterium lactis, Corynebacterium marinum, Corynebacterium maris, Corynebacterium minutissimum, Corynebacterium mustelae, Corynebacterium mycetoides, Corynebacterium phocae, Corynebacterium provencense, Corynebacterium pseudotuberculosis, Corynebacterium renale, Corynebacterium resistens, Corynebacterium riegelii, Corynebacterium simulans, Corynebacterium singulare, Corynebacterium sp. 2183, Corynebacterium sp. 2184, Corynebacterium sp. ATCC 6931, Corynebacterium sp. NML98-0116, Corynebacterium sphenisci, Corynebacterium stationis, Corynebacterium striatum, Corynebacterium terpenotabidum, Corynebacterium testudinoris, Corynebacterium timonense, Corynebacterium ulcerans, Corynebacterium urealyticum, Corynebacterium ureicelerivorans, Corynebacterium uterequi, Corynebacterium variabile, Corynebacterium vitaeruminis, Coxiella burnetii, Crenobacter sp. K1W11S-77, Crinalium epipsammum, Croceibacter atlanticus, Croceicoccus marinus, Croceicoccus naphthovorans, Cronobacter condimenti, Cronobacter dublinensis, Cronobacter malonaticus, Cronobacter muytjensii, Cronobacter sakazakii, Cronobacter turicensis, Cronobacter universalis, Cryobacterium arcticum, Cryobacterium sp. GCJ02, Cryobacterium sp. LW097, Cupriavidus basilensis, Cupriavidus gilardii, Cupriavidus metallidurans, Cupriavidus nantongensis, Cupriavidus necator, Cupriavidus oxalaticus, Cupriavidus pinatubonensis, Cupriavidus sp. USMAA2-4, Cupriavidus taiwanensis, Curtobacterium pusillum, Curtobacterium sp. BH-2-1-1, Curtobacterium sp. MR_MD2014, Curvibacter sp. AEP1-3, Cutibacterium acnes, Cutibacterium avidum, Cutibacterium granulosum, Cyanobacterium aponinum, Cyanobium gracile, Cyanobium sp. NIES-981, Cyanothece sp. ATCC 51142, Cyanothece sp. PCC 7424, Cyanothece sp. PCC 7425, Cyanothece sp. PCC 7822, Cyclobacterium amurskyense, Cyclobacterium marinum, Cycloclasticus zancles, Cylindrospermum stagnale, Cystobacter fuscus, Cytophaga hutchinsonii, Cytophagales bacterium TFI 002, Dactylococcopsis salina, Dechloromonas aromatica, Dechloromonas sp. HYN0024, Deferribacter desulfuricans, Defluviimonas alba, Defluviitoga tunisiensis, Dehalobacter restrictus, Dehalobacterium formicoaceticum, Dehalococcoides mccartyi, Dehalogenimonas formicexedens, Dehalogenimonas lykanthroporepellens, Deinococcus actinosclerus, Deinococcus deserti, Deinococcus ficus, Deinococcus geothermalis, Deinococcus gobiensis, Deinococcus irradiatisoli, Deinococcus maricopensis, Deinococcus peraridilitoris, Deinococcus proteolyticus, Deinococcus puniceus, Deinococcus radiodurans, Deinococcus soli Cha et al. 2016, Deinococcus sp. NW-56, Deinococcus swuensis, Deinococcus wulumuqiensis, Delftia acidovorans, Delftia sp. HK171, Delftia tsuruhatensis, Denitrobacterium detoxificans, Denitrovibrio acetiphilus, Dermabacter vaginalis, Dermacoccus nishinomiyaensis, Dermatophilus congolensis, Desulfallas gibsoniae, Desulfarculus baarsii, Desulfatibacillum alkenivorans, Desulfitobacterium dehalogenans, Desulfitobacterium dichloroeliminans, Desulfitobacterium hafniense, Desulfitobacterium metallireducens, Desulfobacca acetoxidans, Desulfobacter postgatei, Desulfobacterium autotrophicum, Desulfobacula toluolica, Desulfococcus multivorans, Desulfococcus oleovorans, Desulfofarcimen acetoxidans, Desulfohalobium retbaense, Desulfomicrobium baculatum, Desulfomicrobium orale, Desulfomonile tiedjei, Desulfosporosinus acidiphilus, Desulfosporosinus meridiei, Desulfosporosinus orientis, Desulfosporosinus youngiae, Desulfotalea psychrophila, Desulfotomaculum ferrireducens, Desulfotomaculum nigrificans, Desulfotomaculum reducens, Desulfotomaculum ruminis, Desulfovibrio africanus, Desulfovibrio alaskensis, Desulfovibrio desulfuricans, Desulfovibrio fairfieldensis, Desulfovibrio gigas, Desulfovibrio hydrothermalis, Desulfovibrio magneticus, Desulfovibrio piger, Desulfovibrio salexigens, Desulfovibrio sp. FW1012B, Desulfovibrio sp. G11, Desulfovibrio vulgaris, Desulfurella acetivorans, Desulfurispirillum indicum, Desulfurivibrio alkaliphilus, Desulfurobacterium thermolithotrophum, Desulfurococcus amylolyticus, Desulfuromonas soudanensis, Desulfuromonas sp. DDH964, Devosia sp. A16, Devosia sp. H5989, Devosia sp. I507, Devriesea agamarum, Dialister sp. Marseille-P5638, Diaphorobacter polyhydroxybutyrativorans, Dichelobacter nodosus, Dickeya chrysanthemi, Dickeya dadantii, Dickeya dianthicola, Dickeya paradisiaca, Dickeya solani, Dickeya sp. NCPPB 3274, Dickeya zeae, Dictyoglomus thermophilum, Dietzia lutea, Dietzia psychralcaliphila, Dietzia sp. JS16-p6b, Dietzia sp. oral taxon 368, Dietzia timorensis, Dinoroseobacter shibae, Dokdonella koreensis, Dokdonia donghaensis, Dokdonia sp. Dokd-P16, Dokdonia sp. MED134, Dokdonia sp. PRO95, Draconibacterium orientale, Dyadobacter fermentans, Dyella japonica, Dyella jiangningensis, Dyella thiooxydans, Echinicola strongylocentroti, Echinicola vietnamensis, Ectothiorhodospira sp. BSL-9, Edwardsiella hoshinae, Edwardsiella ictaluri, Edwardsiella piscicida, Edwardsiella tarda, Eggerthella lenta, Eggerthella sp. YY7918, Ehrlichia canis, Ehrlichia chaffeensis, Ehrlichia ruminantium, Ehrlichia sp. HF, Elizabethkingia anophelis, Elizabethkingia bruuniana, Elizabethkingia meningoseptica, Elizabethkingia miricola, Elizabethkingia ursingii, Elusimicrobium minutum, Endomicrobium proavitum, Endozoicomonas montiporae, Ensifer adhaerens, Ensifer sojae, Enterobacter asburiae, Enterobacter cancerogenus, Enterobacter cloacae, Enterobacter cloacae complex sp., Enterobacter hormaechei, Enterobacter sp. 638, Enterobacter sp. Crenshaw, Enterobacter sp. FY-07, Enterobacter sp. R4-368, Enterobacter sp. SA187, Enterobacteriaceae bacterium ENNIH1, Enterobacteriaceae bacterium strain FGI 57, Enterococcus casseliflavus, Enterococcus cecorum, Enterococcus durans, Enterococcus faecalis, Enterococcus faecium, Enterococcus gilvus, Enterococcus hirae, Enterococcus mundtii, Enterococcus sp. CR-Ec1, Enterococcus sp. FDAARGOS_375, Enterococcus thailandicus, Enterococcus wangshanyuanii, Entomoplasma luminosum, Entomoplasma melaleucae, Entomoplasma somnilux, Epibacterium mobile, Ereboglobus luteus, Erwinia amylovora, Erwinia billingiae, Erwinia gerundensis, Erwinia persicina, Erwinia tasmaniensis, Erysipelothrix larvae, Erysipelothrix rhusiopathiae, Erysipelotrichaceae bacterium GAM147, Erythrobacter atlanticus, Erythrobacter flavus, Erythrobacter gangjinensis, Erythrobacter litoralis, Erythrobacter seohaensis, Erythrobacter sp. Alg231-14, Erythrobacter sp. HL-111, Erythrobacter sp. KY5, Erythrobacter sp. YH-07, Escherichia albertii, Escherichia coli, Escherichia fergusonii, Escherichia marmotae, Ethanoligenens harbinense, Eubacterium limosum, Euzebya sp. DY32-46, Exiguobacterium antarcticum, Exiguobacterium oxidotolerans, Exiguobacterium sibiricum, Exiguobacterium sp. AT1b, Exiguobacterium sp. MH3, Ezakiella massiliensis, Faecalibacterium prausnitzii, Faecalibaculum rodentium, Faecalitalea cylindroides, Fastidiosipila sanguinis, Fermentimonas caenicola, Ferrimonas balearica, Ferriphaselus amnicola, Fervidobacterium nodosum, Fervidobacterium pennivorans, Fibrella aestuarina, Fibrella sp. ES10-3-2-2, Fibrobacter succinogenes, Fictibacillus arsenicus, Fictibacillus phosphorivorans, Filimonas lacunae, Fimbriimonas ginsengisoli, Finegoldia magna, Fischerella sp. NIES-3754, Fischerella sp. NIES-4106, Flagellimonas sp. HME9304, Flammeovirga sp. MY04, Flammeovirgaceae bacterium 311, Flavisolibacter tropicus, Flavivirga eckloniae, Flavobacteriaceae bacterium, Flavobacteriaceae bacterium 3519-10, Flavobacteriaceae bacterium MAR_2010_188, Flavobacteriaceae bacterium UJ101, Flavobacterium arcticum, Flavobacterium branchiophilum, Flavobacterium columnare, Flavobacterium commune, Flavobacterium crassostreae, Flavobacterium faecale, Flavobacterium gilvum, Flavobacterium indicum, Flavobacterium johnsoniae, Flavobacterium psychrophilum, Flavobacterium sp. AJ004, Flavobacterium sp. CJ74, Flavobacterium sp. HYN0048, Flavobacterium sp. HYN0049, Flavobacterium sp. HYN0056, Flavobacterium sp. HYN0059, Flavobacterium sp. HYN0086, Flavobacterium sp. MEBiC07310, Flavonifractor plautii, Flexistipes sinusarabici, Fluoribacter dumoffii, Fluviicola taffensis, Formosa agariphila, Formosa sp. Hel1_31_208, Formosa sp. Hel1_33_131, Formosa sp. Hel3_A1_48, Francisella halioticida, Francisella noatunensis, Francisella philomiragia, Francisella sp. CA97-1460, Francisella sp. FDC440, Francisella sp. FSC1006, Francisella sp. TX077308, Francisella sp. TX077310, Francisella tularensis, Frankia alni, Frankia casuarinae, Frankia inefficax, Frankia sp. EAN1pec, Frankia sp. QA3, Frankia symbiont of Datisca glomerata, Frankineae bacterium MT45, Frateuria aurantia, Friedmanniella luteola, Friedmanniella sagamiharensis, Frischella perrara, Frondihabitans sp. 762G35, Frondihabitans sp. PAMC 28766, Fuerstia marisgermanicae, Fusarium poae mitovirus 3, Fusobacterium gonidiaformans, Fusobacterium mortiferum, Fusobacterium necrophorum, Fusobacterium ulcerans, Fusobacterium varium, Gallaecimonas sp. HK-28, Gallibacterium anatis, Gallionella capsiferriformans, Gammaproteobacteria bacterium DM2, Gammaproteobacteria bacterium ESL0073, Gardnerella vaginalis, Geitlerinema sp. PCC 7407, Gemella sp. ND 6198, Gemella sp. oral taxon 928, Geminocystis herdmanii, Geminocystis sp. NIES-3708, Geminocystis sp. NIES-3709, Gemmata obscuriglobus, Gemmata sp. SH-PL17, Gemmatimonas aurantiaca, Gemmatimonas phototrophica, Gemmatirosa kalamazoonesis, Gemmobacter sp. HYN0069, Geoalkalibacter subterraneus, Geobacillus genomosp. 3, Geobacillus lituanicus, Geobacillus sp. WCH70, Geobacillus stearothermophilus, Geobacillus subterraneus, Geobacillus thermocatenulatus, Geobacillus thermodenitrificans, Geobacillus thermoleovorans, Geobacter anodireducens, Geobacter bemidjiensis, Geobacter daltonii, Geobacter lovleyi, Geobacter metallireducens, Geobacter pickeringii, Geobacter sp. DSM 9736, Geobacter sp. M18, Geobacter sp. M21, Geobacter sulfurreducens, Geobacter uraniireducens, Geodermatophilus obscurus, Geoglobus ahangari, Geosporobacter ferrireducens, Gibbsiella quercinecans, Gilliamella apicola, Gillisia sp. Hel1_33_143, Gilvibacter sp. SZ-19, Glaciecola nitratireducens, Glaesserella parasuis, Glaesserella sp. 15-184, Gloeobacter kilaueensis, Gloeobacter violaceus, Gloeocapsa sp. PCC 7428, Gloeomargarita lithophora, Gluconacetobacter diazotrophicus, Gluconobacter albidus, Gluconobacter oxydans, Glutamicibacter arilaitensis, Gordonia bronchialis, Gordonia iterans, Gordonia phthalatica, Gordonia polyisoprenivorans, Gordonia rubripertincta, Gordonia sp. 1D, Gordonia sp. KTR9, Gordonia sp. YC-JH1, Gordonia terrae, Gordonibacter massiliensis, Gordonibacter pamelaeae, Gordonibacter urolithinfaciens, Gottschalkia acidurici, Gramella flava, Gramella forsetii, Gramella salexigens, Gramella sp. MAR_2010_102, Gramella sp. MAR_2010_147, Gramella sp. SH35, Granulibacter bethesdensis, Granulicella mallensis, Granulicella tundricola, Granulosicoccus antarcticus, Grimontia hollisae, Gynuella sunshinyii, Haematospirillum jordaniae, Haemophilus aegyptius, Haemophilus haemolyticus, Haemophilus influenzae, Haemophilus pittmani* | 3289 |
| Healthy + Diabetic_Periodontitis + Advanced_Periodontitis | *Actinomyces hongkongensis, Aggregatibacter aphrophilus, Aggregatibacter segnis, Filifactor alocis, Haemophilus parainfluenzae, Lachnospiraceae bacterium oral taxon 500, Leptotrichia sp. oral taxon 212, Neisseria elongata, Neisseria meningitidis, Neisseria mucosa, Neisseria sicca, Neisseria subflava, Streptococcus cristatus, Streptococcus sanguinis* | 14 | *Bifidobacterium catenulatum, Bifidobacterium longum, Candidatus Saccharibacteria oral taxon TM7x, Cryptobacterium curtum, Lactobacillus mucosae, Olsenella umbonata, Parascardovia denticolens* | 7 |
| Healthy only | *Actinomyces gaoshouyii, Actinomyces sp. 2129, Atopobium parvulum, Lactobacillus plantarum, Streptococcus agalactiae, Streptococcus mutans, Streptococcus pseudopneumoniae, Streptococcus sp. oral taxon 431, Streptococcus thermophilus* | 9 | *Anaerolineaceae bacterium oral taxon 439, Streptococcus pyogenes* | 2 |
| Diabetic_Periodontitis + Advanced_Periodontitis | *Desulfobulbus sp. ORNL, Lachnoanaerobaculum umeaense, Treponema putidum* | 3 | *Actinomyces meyeri, Bifidobacterium dentium, Lactobacillus crispatus, Lactobacillus fermentum, Lactobacillus gasseri, Rothia mucilaginosa, Streptococcus salivarius* | 7 |
| Diabetic_Periodontitis only | *Capnocytophaga sp. oral taxon 878* | 1 | *Actinomyces sp. Chiba101, Capnocytophaga ochracea, Capnocytophaga sp. oral taxon 864, Fusobacterium sp. oral taxon 203, Gemella morbillorum, Olsenella uli, Prevotella dentalis, Streptococcus constellatus* | 8 |
| Advanced_Periodontitis only | *Mogibacterium pumilum, Neisseria gonorrhoeae* | 2 | *Campylobacter concisus, Dialister pneumosintes, Mogibacterium diversum, Prevotella fusca, Prevotella jejuni, Prevotella scopos, Streptococcus parasanguinis* | 7 |
| Healthy + Early_Periodontitis | *Actinomyces meyeri, Bifidobacterium dentium, Lactobacillus crispatus, Lactobacillus fermentum, Lactobacillus gasseri, Rothia mucilaginosa, Streptococcus salivarius* | 7 | *Desulfobulbus sp. ORNL, Lachnoanaerobaculum umeaense, Treponema putidum* | 3 |
| Healthy + Advanced_Periodontitis | *Eikenella corrodens* | 1 |  | 0 |
| Healthy + Early_Periodontitis + Advanced_Periodontitis | *Actinomyces sp. Chiba101, Capnocytophaga ochracea, Capnocytophaga sp. oral taxon 864, Fusobacterium sp. oral taxon 203, Gemella morbillorum, Olsenella uli, Prevotella dentalis, Streptococcus constellatus* | 8 | *Capnocytophaga sp. oral taxon 878* | 1 |
| Early_Periodontitis + Advanced_Periodontitis + Diabetic_Periodontitis | *Anaerolineaceae bacterium oral taxon 439, Streptococcus pyogenes* | 2 | *Actinomyces gaoshouyii, Actinomyces sp. 2129, Atopobium parvulum, Lactobacillus plantarum, Streptococcus agalactiae, Streptococcus mutans, Streptococcus pseudopneumoniae, Streptococcus sp. oral taxon 431, Streptococcus thermophilus* | 9 |
| Early_Periodontitis+ Diabetic_Periodontitis |  | 0 | *Eikenella corrodens* | 1 |
| Early_Periodontitis only | *Bifidobacterium catenulatum, Bifidobacterium longum, Candidatus Saccharibacteria oral taxon TM7x, Cryptobacterium curtum, Lactobacillus mucosae, Olsenella umbonata, Parascardovia denticolens* | 7 | *Actinomyces hongkongensis, Aggregatibacter aphrophilus, Aggregatibacter segnis, Filifactor alocis, Haemophilus parainfluenzae, Lachnospiraceae bacterium oral taxon 500, Leptotrichia sp. oral taxon 212, Neisseria elongata, Neisseria meningitidis, Neisseria mucosa, Neisseria sicca, Neisseria subflava, Streptococcus cristatus, Streptococcus sanguinis* | 14 |
| Early_Periodontitis + Advanced_Periodontitis |  | 0 | *Neisseria sp. KEM232, Rothia dentocariosa, Streptococcus sobrinus* | 3 |
| Healthy + Early_Periodontitis + Diabetic_Periodontitis |  | 0 | *Mogibacterium pumilum, Neisseria gonorrhoeae* | 2 |

Supplementary Figure S1: Hierarchical treemaps of the top 50 pathways by abundance in subgingival plaque for each clinical group: healthy (top-left), early periodontitis (top-right), advanced periodontitis (bottom-left), and diabetic periodontitis (bottom-right). Each tile is a pathway (pathway name); tile area is proportional to group-level raw abundance. Tiles are grouped by and color-coded to broad function (identical palette across panels); watermark text inside each block shows the broad function. Overlaid pathway labels display the pathway name and abundance (labels may be abbreviated for very small tiles like advanced periodontitis).
